# Using health technology assessment to assess the value of new medicines: results of a systematic review and expert consultation across eight European countries

**DOI:** 10.1007/s10198-017-0871-0

**Published:** 2017-03-16

**Authors:** Aris Angelis, Ansgar Lange, Panos Kanavos

**Affiliations:** Department of Social Policy and Medical Technology Research Group, LSE Health, London School of Economics and Political Science, Houghton Street, London, WC2A 2AE UK

**Keywords:** Health technology assessment (HTA), Value assessment, Innovative medicines, High cost medicines, Pharmaceutical policy, European Union, Systematic review, Expert consultation, I (Health, Education, and Welfare), I1 (Health), I10 (General), I11 (Analysis of Health Care Markets), I18 (Government Policy; Regulation; Public Health)

## Abstract

**Background:**

Although health technology assessment (HTA) systems base their decision making process either on economic evaluations or comparative clinical benefit assessment, a central aim of recent approaches to value measurement, including value based assessment and pricing, points towards the incorporation of supplementary evidence and criteria that capture additional dimensions of value.

**Objective:**

To study the practices, processes and policies of value-assessment for new medicines across eight European countries and the role of HTA beyond economic evaluation and clinical benefit assessment.

**Methods:**

A systematic (peer review and grey) literature review was conducted using an analytical framework examining: (1) ‘Responsibilities and structure of HTA agencies’; (2) ‘Evidence and evaluation criteria considered in HTAs’; (3) ‘Methods and techniques applied in HTAs’; and (4) ‘Outcomes and implementation of HTAs’. Study countries were France, Germany, England, Sweden, Italy, Netherlands, Poland and Spain. Evidence from the literature was validated and updated through two rounds of feedback involving primary data collection from national experts.

**Results:**

All countries assess similar types of evidence; however, the specific criteria/endpoints used, their level of provision and requirement, and the way they are incorporated (e.g. explicitly vs. implicitly) varies across countries, with their relative importance remaining generally unknown. Incorporation of additional ‘social value judgements’ (beyond clinical benefit assessment) and economic evaluation could help explain heterogeneity in coverage recommendations and decision-making.

**Conclusion:**

More comprehensive and systematic assessment procedures characterised by increased transparency, in terms of selection of evaluation criteria, their importance and intensity of use, could lead to more rational evidence-based decision-making, possibly improving efficiency in resource allocation, while also raising public confidence and fairness.

**Electronic supplementary material:**

The online version of this article (doi:10.1007/s10198-017-0871-0) contains supplementary material, which is available to authorized users.

## Background

Current value assessment and appraisal approaches of medical technologies using economic evaluation or adopting comparative clinical benefit assessment in order to inform coverage decisions and improve efficiency in resource allocation have been subject to criticism for a number of reasons.

Most health technology assessment (HTA) systems base their decision-making process on cost per outcome metrics of economic evaluations such as, for example, the cost per quality adjusted life year (QALY) [1]. However a key limitation of the QALY approach is the inadequacy of capturing social value [2–4]. It is clear that a central aim of more recent approaches to value measurement, including value-based assessment and value-based pricing, involves the incorporation of additional parameters capturing other dimensions of value into the overall valuation scheme [5, 6]. Although a number of additional criteria beyond scientific value judgements are considered to assess the evidence submitted and inform coverage decisions in different HTA settings [7], their use remains implicit or ad hoc rather than explicit and systematic.

Another drawback is caused by the way in which value is assessed and appraised, often resulting in unexplained heterogeneity of coverage decisions across settings even for the same drug-indication pair [8–14]. Although some of this decision heterogeneity could be justified on the grounds of different budget constraints and national priorities, inconsistencies in medicines’ eligibility for reimbursement across countries can give rise to an international ‘post-code’ lottery for patient access, even in the same geographical region and can have important implications for equity and fairness, especially when differences remain unexplained [11]. Several studies have acknowledged the need for well-defined decision-making processes that are fairer and more explicit [15–17]. By ensuring ‘accountability for reasonableness’ and providing a better understanding of the rationale behind decision-making, decisions will also have enhanced legitimacy and acceptability [12, 18].

By reviewing and synthesising the evidentiary requirements (both explicit and implicit), the methods and techniques applied and how they contribute to decision-making, the objective of this study is to provide a critical review of value assessment and appraisal methods for new medicines, including the evaluation criteria employed across a number of jurisdictions in Europe deploying explicit evaluation frameworks in their HTA processes. More specifically, the study seeks to determine whether HTA processes incorporate additional criteria beyond economic evaluation or clinical benefit assessment, and, if so, which ones and how they inform coverage recommendations. To date no study has provided a similar review and analysis of HTA policies and practices for innovative medicines across different European countries to this extent. In fulfilling the above aims, the next section outlines the methods and includes the components of the analytical framework adopted for this purpose; subsequently, the evidence collected from eight European countries is presented and discussed, before presenting the policy implications.

## Methods

We outline and propose a conceptual framework to facilitate the systematic review of HTA processes and capture their salient features across settings following previous evidence [19]. Based on that, we collected the relevant evidence, relying on both primary and secondary sources. The evidence base covered eight EU Member States that have arms-length HTA agencies and recognised HTA processes. The study took place in the context of Advance-HTA, an EU-funded project aiming to contribute to advances in the methods and practices for HTA in Europe and elsewhere [20].

Secondary sources of evidence comprised a systematic review of the country-specific value-assessment peer review literature using an analytical framework to investigate the practices, processes and policies of value-assessment and their impact, as observed in the study countries.

Evidence from the literature was validated by means of two rounds of feedback involving primary data collection: the first was from Advance-HTA consortium partners [20], while the second involved a detailed validation of the study’s results by national experts following the incorporation of all literature results and feedback from Advance-HTA partners.

### Analytical framework outlining the value assessment and appraisal characteristics of HTA systems

Existing frameworks for analysing and classifying coverage decision-making systems for health technologies were reviewed and adjusted according to the needs of the current examination, which focuses on the assessment and appraisal stages of the coverage review procedure from the HTA agency’s or institution’s point of view, without having any special interest on the decision outcomes per se [21–23].

The main value assessment and appraisal characteristics necessary to outline the practices and processes in the different countries of interest as reflected through their national HTA agencies were classified using an analytical framework consisting of four key components, each having a number of different sub-components: (1) ‘Responsibilities and structure of HTA agencies’; (2) ‘Evidence and evaluation criteria considered in HTAs’; (3) ‘Methods and techniques applied in HTAs’; and (4) ‘Outcomes and implementation of HTAs’. These were considered to be the main components needed in order to sufficiently capture the features of the different HTA systems.

**Fig. 1:**
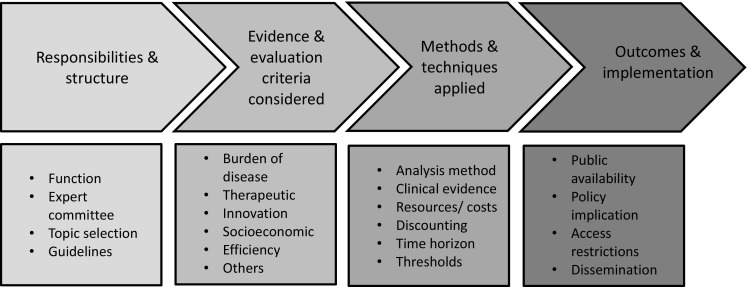
Main components and sub-components of the analytical framework applied

In the context of this study, the second component was more extensively examined because a key subject of our investigation was to identify and analyse any additional concerns and evaluation criteria beyond those informing economic evaluations or clinical benefit assessment. The sub-components of the main components are described below and are shown in Fig. 1.

#### Responsibilities and structure of HTA agencies

The first component considers the operational characteristics of national HTA agencies. It includes details about the function and responsibilities of HTA agencies, the relevant committees within agencies tasked with assessment and appraisal, details on the topic selection process, and whether methodological guidelines exist for the conduct of pharmacoeconomic analysis.

#### Evidence and evaluation criteria considered in HTAs

This component relates to the types of evidence evaluated and the particular evaluation criteria considered. Generally, the assessed evidence can be classified into features relating to the disease (indication) under consideration, or into characteristics relating to the technology being assessed. The former is reflected through the ‘burden of disease’ (BoD), i.e. the impact that the disease has, which depends mainly on the severity of the disease and the unmet medical need. The latter can be classified into clinical benefit (mainly therapeutic impact and safety considerations), innovation (e.g. clinical novelty and nature of treatment), and socioeconomic impact (e.g. public health impact, productivity loss impact). Other important characteristics relate to efficiency (e.g. cost-effectiveness, cost), ethical/equity considerations, accepted data sources, and relative importance (i.e. weighting) of the evidence.

#### Methods and techniques applied in HTAs

This component is associated with the evaluation methods and techniques used. In terms of the analytical methods applied (i.e. comparative efficacy/effectiveness, type of economic evaluation), methodologies differ based on their outcome measure and their elicitation technique, the choice of comparator(s) and the perspective adopted. In relation to the clinical evidence used to populate the analysis, crucial details involve accepted or preferred data sources (i.e. study designs), data collection approaches (e.g. requirement for systematic literature reviews) and synthesis (e.g. suggestion for meta-analysis) of the data. In terms of resources used, important considerations include the types of costs and data sources. For both clinical outcomes and costs, discount rate(s) applied and time horizons assumed are included, together with the existence of any explicit or implicit willingness-to-pay (WTP) thresholds on cost-effectiveness based on which recommendations are made.

#### Outcomes and implementation of HTAs

The final component relates to the outcomes of the evaluation procedures and their implementation. Key characteristics include the public availability of the evaluation report; the policy implications of whether and how outcomes are applied in practice (e.g. pricing vs. reimbursement); the usage of any access restrictions; how decisions are disseminated and implemented; whether appeal procedures are available; and the frequency of any recommendation revisions.

### Systematic literature review

The systematic literature review methodology was based on the Centre for Reviews and Dissemination (CRD) guidance for undertaking systematic reviews in health care [24].

#### Inclusion criteria (country selection and study period)

The study countries (and the respective HTA agencies) were France (Haute Autorité de Santé, HAS), Germany (Institut für Qualität und Wirtschaftlichkeit im Gesundheitswesen, IQWiG), Sweden (Tandvårds- och läkemedelsförmånsverket, TLV), England (National Institute for Health and Care Excellence, NICE), Italy^1^ (Agenzia Italiana del Farmaco, AIFA), the Netherlands [Zorginstituut Nederland, ZIN (formerly College voor zorgverzekeringen, CVZ)], Poland (The Agency for Health Technology Assessment and Tariff System, AOTMiT) and Spain [Red de Agencias de Evaluación de Tecnologías Sanitarias y Prestaciones del Sistema Nacional de Salud (RedETS) and the Interministerial Committee for Pricing (ICP)].^2^ The study countries were selected because of their variation in health system financing (tax-based vs. social insurance-based), the organisation of the health care system (central vs. regional organisation), the type of HTA in place (predominantly economic evaluation vs. predominantly clinical benefit assessment), and the perspective used in HTA (health system vs. societal), so that the sample is representative of different health systems and HTA approaches across Europe.

The study period for inclusion of relevant published studies was from January 2000 to January 2014, with article searches taking place in February 2013 in the first instance and an update taking place at the end of January 2014. The year of 2000 was selected as the start date because the HTA activity of most countries started then or was significantly expanded in scope since then. Feedback from the Advance-HTA consortium partners was provided in August 2014. Additional input, including the most recent updates on national HTA processes, was collected from HTA experts and national competent authorities between March and May 2016.

#### Identification of evidence

Two electronic databases (MEDLINE—through PubMed resource—and the Social Science Citation Index—through the Web of Science portal) were searched for peer-reviewed literature only using a search strategy for English articles published up until the time of the literature search (including all results from the oldest to the latest available) using the following keywords: ‘health technology assessment + pharmaceuticals’; ‘health technology assessment + methodologies’; ‘value assessment + pharmaceuticals’; and ‘value assessment + methodologies’. Furthermore, reference lists from the studies selected were screened (see following section), retrieving any additional studies cited that could be of relevance. Finally, grey literature was searched including published guidelines from the HTA agencies available online through each agency’s website.

#### Study selection and data extraction

**Fig. 2:**
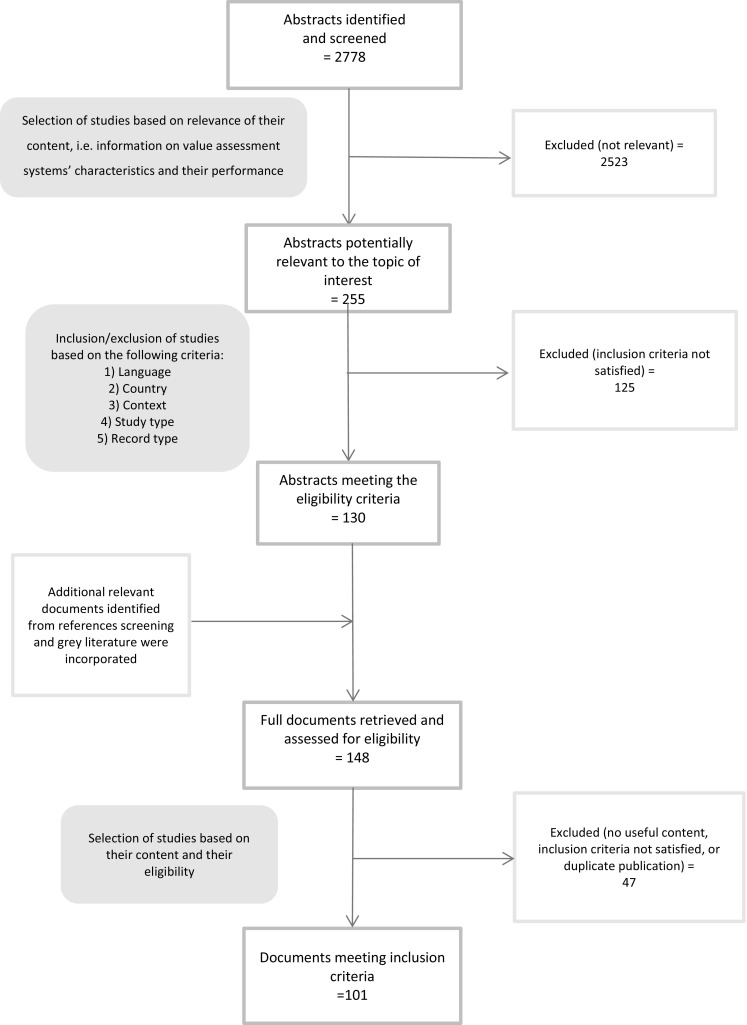
Flow chart of literature review process

Articles were selected according to a four-stage process as outlined in Fig. 2 [24]. In the first stage, all titles and abstracts were reviewed, with abstracts not relevant to the topic excluded; where content relevance could not be determined, articles were passed through to the next stage. In the second stage, all relevant abstracts were assessed against a number of pre-determined selection criteria by two of the authors; these criteria included: (1) language (only English articles were included), (2) study country (only studies examining the eight countries of interest were included), (3) study context (only national coverage HTA perspectives were included), (4) study type (product-specific technology appraisal reports were excluded), (5) record type (conference proceedings or titles with no abstracts available were excluded). In the third stage, full articles for all abstracts meeting the eligibility criteria were retrieved; in addition, relevant studies identified from reference screening and grey literature, including published guidelines from HTA agencies, were incorporated (non-English articles cited by English documents were included in this stage). Finally, in the fourth stage, full articles were reviewed and relevant data were extracted. An Excel template listing the value assessment and appraisal characteristics (categories and sub-categories) of interest was used for data extraction. Data were extracted in free text form, with no limitations on the number of free text fields, and as little categorisation of data as possible, in order to avoid loss of information. The lead author extracted the data while the other authors independently checked the extracted templates for completeness and accuracy.

### Expert consultation

Upon consultation of the preliminary results with the partners of the Advance-HTA consortium, it became obvious that in a few cases (primarily for France and to a lesser degree for Sweden), the evidence from the peer review literature was outdated and did not reflect actual practices, being even contradictory in some cases. As a result, we solicited comments and feedback from the consortium partners in order to update and supplement the information extracted from the systematic review. In a final step, all updated results tables were shared with HTA experts in the study countries, who were asked to review and validate the outputs of the study. Experts (*n* = 18) were affiliated with academic or research institutions (36% of total) and national competent authorities, such as HTA agencies or payer bodies (64% of total), and provided further evidence and guidance, including—in some cases—additional literature sources outside the originally selected review period, if appropriate. Expert input from these two rounds of consultation are quoted as ‘personal communication’ from the Advance-HTA project [25].

## Results

Figure 2 shows a flow chart of the review process and the respective number of articles in each stage. In total, 2778 potentially eligible peer-reviewed article listings were identified in the electronic databases; of these 255 articles were identified as potentially useful and were read in full. A total of 130 articles met the eligibility criteria, and an additional 18 articles were identified as possibly relevant through reference screening or as grey literature. The content of 101 articles from the literature review was finally used to inform the findings (Supplementary Appendix 1). An additional five studies were identified during the expert consultation process and were taken into consideration in discussing and interpreting the results (Supplementary Appendix 2).

### Responsibilities and structure of national HTA agencies

Across the study countries, HTA agencies exist mainly in the form of autonomous governmental bodies, having either an advisory or regulatory function. Usually, a technical group is responsible for early assessment of the evidence following which an expert committee appraises the request for coverage and produces recommendation(s) for the final decision body.

The topic selection process is generally not entirely transparent, with the belief that most agencies predominantly assess new medical technologies that are expensive and/or with uncertain benefits. In some cases, topic selection is not applicable as all technologies that apply for reimbursement need to be assessed.

In all study countries, with the exception of Italy and Spain, official country-specific pharmacoeconomic guidelines for the evaluation process are available, mainly concerning methodological and reporting issues [26, 27]. In England, in addition to the evaluation process, guidelines also exist for the purpose of application submission requirements, including the description of key principles of the appraisal methodology adopted by NICE [27]. For all countries, application of the guidelines is recommended. It is worth clarifying that although some of the HTA agencies tend to focus on medicines, others evaluate all types of health care interventions; in this case the term “pharmacoeconomic” might not be adequately representative of the types of guidelines in place, in which case they could be referred to as “methods for HTA” as in the case of NICE. A summary of the responsibilities and structure of the national HTA agencies in the study countries is presented in Table 1.

**Table 1:** Responsibilities and structure of national heath technology assessment (HTA) agencies

	France (HAS/CEESP)	Germany (IQWiG)	Sweden (TLV)	England (NICE)	Italy (AIFA)	Netherlands (ZIN)	Poland (AOTMiT)	Spain (RedETS/ISCIII or ICP^a^)
Function	Autonomous, advisory	Autonomous, advisory	Autonomous, regulatory	Autonomous, advisory	Autonomous, regulatory	Autonomous, advisory	Autonomous, advisory	Autonomous, advisory
Expert committee	CEESP	Assessment: IQWiG scientific personnel^b^; Appraisal: G-BA	The Board for Pharmaceutical Benefits	Technology Appraisal Committee	AIFA’s Technical Scientific Committee and CPR	Committee for societal consultation regarding the benefit basket	Transparency Council	ICP^c^
Topic selection	HAS (about 90% submitted by the manufacturers, 10% requested by the MoH)^d^	Not applicable (all drugs applying for marketing authorization, excluding inpatient)	TLV (only outpatient and high price drugs)	DH in consultation with NICE based on explicit prioritisation criteria^e^	AIFA (all drugs submitted by manufacturers)	Mostly on its own initiative; sometimes at the request of MoH	MoH^f^ (in the case of manufacturer submission—triggered by MAH)	Not subject to any specific known procedure^g^
Guidelines for the conduct of economic analysis	Yes	Yes (however, CBA is not standard practice)	Yes	Yes	In progress	Yes	Yes	Spanish recommendations on economic evaluation of health technologies

*Source* The authors (based on literature review findings and expert consultation)

* HAS* Haute Autorité de Santé,* CEESP* Transparency Commission, Economic Evaluation and Public Health Commission,* IQWiG* Institut für Qualität und Wirtschaftlichkeit im Gesundheitswesen,* TLV* Tandvårds- och läkemedelsförmånsverket,* NICE* National Institute for Health and Care Excellence,* AIFA* Agenzia Italiana del Farmaco,* ZIN* Zorginstituut Nederland,* AOTMiT* Agency for Health Technology Assessment and Tariff System,* RedETS* Red de Agencias de Evaluación de Tecnologías Sanitarias y Prestaciones del Sistema Nacional de Salud,* ICP* Interministerial Committee for Pricing,* MoH* Ministry of Health,* MAH* market authorisation holder,* DH* Department of Health,* CBA* cost benefit analysis,* G-BA* Federal Joint Committee (Gemeinsame Bundesausschuss),* CPR* AIFA’s Pricing and Reimbursement Committee

^a^RedETS is the Spanish Network of regional HTA agencies, coordinated by the Institut de Salud Carlos III (ISCIII), responsible for the evaluation of non-drug health technologies. The ICP, led by the Dirección General de Farmacia under the Ministry of Health, is the committee responsible for the evaluation of drugs producing mandatory decisions at national level

^b^For orphans, assessment is also done by the G-BA

^c^The ICP involves representatives from the Ministry of Health, Ministry of Industry, and Ministry of Finance together with a dynamic (i.e. rotating) set of expert representatives from the autonomous communities

^d^An economic evaluation is performed only for a subset of new products meeting certain criteria (manufacturer claims a high added value/product is likely to have a significant impact on public health expenditures)

^e^Criteria include expected health benefit, population size, disease severity, resource impact, inappropriate variation in use and expected value of conducting a NICE technology appraisal

^f^Regulated by law: the Act of 27 August 2004 on healthcare benefits financed from public funds; the Act of 12 May 2011 on the reimbursement of medicinal products, special purpose dietary supplements and medical devices

^g^For new drugs, manufacturers have to submit a dossier for evaluation when they apply for pricing and reimbursement. Topic selection for non-drug technologies under the action of RedETS is well developed with the participation of informants from all autonomous communities based on a two round consultation

### Evidence and evaluation criteria considered in HTAs

Generally all countries assess the same groups of evidence, however the individual parameters considered and the way they are evaluated differ from country to country. All countries acknowledge the consideration of a wide variety of data sources including scientific studies (e.g. clinical trials, observational studies), national statistics, clinical practice guidelines, registry data, surveys, expert opinion and other evidence from pharmaceutical manufacturers [28]. A summary of the evidence and the evaluation criteria under consideration across the study countries is presented in Table 2.

**Table 2:** Evidence and evaluation criteria considered in HTAs

	France (HAS/CEESP)	Germany (IQWiG)	Sweden (TLV)	England (NICE)	Italy (AIFA)	Netherlands (ZIN)	Poland (AOTMiT)	Spain (RedETS/ISCIII or ICP)
**Burden of disease**								
Severity	Yes, as part of SMR	Yes, as part of added benefit assessment	Yes (impact on WTP threshold)^a^	Yes (mainly as part of EoL treatments)	Yes (implicitly)	Yes^b^	Yes^c^	Yes
Availability of treatments (i.e. unmet need)	Yes (binary: Yes/No)	True for other technologies rather than pharmaceuticals^d^	Yes, indirectly (captured by severity)	Yes (clinical need as a formal criterion)	Yes^e^	Yes^f^	Yes^g^	Yes
Prevalence (e.g. rarity)	Yes, informally	As part of G-BA’s decision-making process^h^	Yes	Yes	Yes^I^	Yes	Yes^j^	Yes
**Therapeutic and safety impact**								
Efficacy	Yes (4 classifications via SMR, 5 via ASMR)^k^	Yes (6 classifications)^l^	Yes	Yes	Yes	Yes	Yes^m^	Yes
Clinically meaningful outcomes	Yes (preferred)	Yes (preferred)	Yes	Yes (preferred)	Yes	Yes	Yes^n^	Yes
Surrogate/intermediate outcomes	Considered	Considered	Considered	Considered	Considered	Considered	Considered^o^	Considered
HRQoL outcomes	Generic; disease-specific	Generic; disease-specific^p^	Generic (preferred); disease-specific	Generic; disease-specific	Generic; disease-specific	Yes	Yes^q^	Yes (including patient well-being)
Safety	Yes	Yes^r^	Yes	Yes	Yes	Yes	Yes^s^	Yes
Dealing with uncertainty	Implicitly (preference for RCTs), explicitly (robustness of evidence)	Explicitly (classification of empirical studies and complete evidence)	Implicitly (through preference for RCTs)	Explicitly (quality of evidence), implicitly (preference for RCTs), indirectly (rejection if not scientifically robust)	Yes, registries and MEAs are used to address uncertainty	Implicitly (if included in the assessment studies)	No^t^	Can be considered as part of economic evaluation
**Innovation level**								
Clinical novelty	Yes (as part of ASMR) if efficacy/safety ratio is positive	Implicitly as part of added therapeutic benefit consideration^u^	Yes, but only if it can be captured in the CE analysis	Yes	Yes	Yes	Yes^v^	Yes^w^
Ease of use and comfort	Not explicitly, in some cases^x^	Only if relevant for morbidity/side effects, not explicitly considered for benefit assessment^y^	Yes (to some extent)	Not explicitly	No	Not standard, case-by-case basis	No^z^	Not explicitly, indirectly^aa^
Nature of treatment/technology	Yes (3 classifications)^ab^	Not explicitly considered for benefit assessment	Not explicitly	Yes (when above £20,000)	No	Implicitly	Yes^ac^	Yes (through the degree of innovation criterion)
**Socio-economic impact**								
Public health benefit/value	Yes, rarely via “intérêt de Santé Publique”^ad^	No^ae^	Yes, indirectly^af^	As indicated in guidance to NICE to be considered in the evaluation process^ag^	Implicitly	Yes (explicit estimates)	Yes^ah^	Social utility of the drug and rationalisation of public drug expenditures
Social productivity	Not explicitly^ai^	Yes^aj^	Indirect costs considered explicitly (to some extent)	Productivity costs excluded but informal “caregiving” might be considered	Direct costs only^ak^	Yes	No^al^	Yes, either explicitly or implicitly
**Efficiency considerations**								
Cost-effectiveness	Yes^am^	Optional (cost-benefit)^an^	Yes (cost-efficiency as a principle)	Yes	Yes	Yes	Yes, mandatory by law	Yes (not mandatory)
CBA/BIA	Not mandatory but BIA is highly recommended^ao^	BIA (mandatory)	Cost only considered for treatments of the same condition; BIA not mandatory	BI to NHS, PSS, hospitals, primary care	Yes	Yes	Yes, payer affordability mandatory by law	Yes (BI to NHS)
**Other evidence and criteria**								
Place in therapeutic strategy	Yes^ap^	Evaluation usually specifies the line of treatment	Evaluation usually specifies the line of treatment	Broad clinical priorities for the NHS (by Secretary of State)	Yes	Not explicitly	No	Yes^aq^
Conditions of use	Yes (e.g. the medicine is assessed in each of its indications, if several)	No, drug is in principle reimbursable for the whole indication spectrum listed on its authorisation^ar^	Yes, coverage can be restricted based on evidence at sub-population level	Yes, coverage can be restricted based on evidence at sub-population level	Implicitly	Yes, indications	Yes, coverage can be restricted to strictly defined sub-populations	Yes (several medicines are introduced with Visado—Prior Authorization Status)
Ethical considerations	Not incorporated in assessment^as^	Sometimes (implicitly)	Yes	Yes^at^	Implicitly	Yes, explicitly (e.g. solidarity and affordability)^au^	Considered on the basis of HTA Guidelines	Not explicitly
Weights/relative importance of different criteria	Not transparent	Not transparent	“Human dignity” usually being overriding^av^	Not transparent	Not transparent	Therapeutic value is the most important criterion	Not transparent	Not transparent and not consistent across regions^aw^
Accepted data sources (for estimating number of patients, clinical benefits and costs)	Clinical trials, observational studies, national statistics, clinical guidelines, surveys, expert opinions	RCTs^ax^, national or local statistics, clinical guidelines, surveys, price lists, expert opinions (including patient representatives)	Clinical trials, observational studies, national statistics, clinical guidelines, surveys, expert opinions	Clinical trials, observational studies, national or local statistics, clinical guidelines, surveys, expert opinions	Clinical trials, observational studies, national statistics, clinical guidelines, surveys, expert opinions, scientific societies‘ opinion	Clinical trials, clinical guidelines, expert opinions	Clinical trials, observational studies, national or local statistics, clinical guidelines, surveys, expert opinions	Clinical trials, observational studies, national statistics, clinical guidelines, surveys, expert opinions

*Source* The authors (based on literature review findings and expert consultation)

* SMR* Service Médical Rendu,* ASMR* Amélioration du Service Médical Rendu,* RCT* randomised clinical trial,* HRQoL* health-related quality of life,* MEA* managed entry agreement,* EoL* end of life,* WTP* willingness to pay,* BIA* budget impact analysis,* NHS* National Health System,* PSS* personal social services

^a^Severity can be defined on the basis of several elements of the condition, including the risk of permanent injury and death

^b^Both explicitly and implicitly; more recently they tend to explicitly take into account “burden of disease” measures

^c^Regulated by law: the Act of 27 August 2004 on healthcare benefits financed from public funds

^d^In evaluations performed by the G-BA to determine the benefit basket (i.e. not drugs, which are covered automatically after marketing authorization and value assessment plays a role for the price) availability or lack of alternatives and the resulting medical necessity are considered to determine clinical benefit

^e^Explicitly stated in the legislation as a criterion to set price

^f^Estimate the number of treatments that is considered necessary and compared that with the actual capacity

^g^Not obligatory by law; considered in the assessment process of AOTMiT on the basis of HTA guidelines (good HTA practices)

^h^Lower accepted significance levels for *P* values (e.g. 10% significance levels) for small sample sizes such as rare disease populations; acceptance of evidence from surrogate endpoints rather than only ‘hard’ or clinical endpoints

^I^Decisions on price and reimbursement of orphan drugs are made through a 100-day ad-hoc accelerated procedure, although criteria for HTA appraisals do not differ from non-orphan drugs

^j^Commonness, but not rarity, regulated by law (the Act on healthcare benefits); rarity is considered in the assessment process in AOTMiT on the basis of HTA guidelines

^k^SMR, 4 classifications for actual clinical benefit: Important/High (65% reimbursement rate), Moderate (30%), Mild/Low (15%), Insufficient (not included on the positive list); ASMR, 5 classifications for relative added clinical value: Major (ASMR I), Important (ASMR II), Moderate (ASMR III), Minor (ASMR IV), No clinical improvement (ASMR V)

^l^The possible categories are: major added benefit, considerable added benefit and minor added benefit. Three additional categories are recognized: non-quantifiable added benefit, no added benefit, and lesser benefit

^m^Regulated by law: the Act of 27 August 2004 on healthcare benefits financed from public funds

^n^Regulated by law: the Act on the reimbursement

^o^Weak preference; if no LYG/QALY data available

^p^Considered if measured using validated instruments employed in the context of clinical trials

^q^Regulated by law: the Act on reimbursement

^r^Based on the following ranking relative to comparator: greater harm, comparable harm, lesser harm

^s^Regulated by law: the act on healthcare benefits; the act on reimbursement

^t^Not obligatory by law; considered in the assessment process of AOTMiT on the basis of HTA guidelines (good HTA practices)

^u^Not a criterion per se, implicitly considered if patient benefit is higher than that of existing alternatives

^v^The Act on healthcare benefits considers the following classifications: saving life and curative, saving life and improving outcomes, preventing premature death, improving HRQoL without life prolongation

^w^Incremental clinical benefit is considered as part of the therapeutic and social usefulness criterion

^x^Only considered in the ASMR if it has a clinical impact (e.g. through a better compliance)

^y^The IQWiG’s general methodology (not specifically for new drugs) states that patient satisfaction can be considered as an additional aspect, but it is not adequate as a sole deciding factor

^z^Not obligatory by law (unless captured in HRQoL/QALY); considered in the assessment process of AOTMiT on the basis of HTA guidelines

^aa^Through the therapeutic and social usefulness criterion

^ab^Ranking includes the following classifications: Symptomatic relief, Preventive treatment, Curative therapy

^ac^Regulated by law: the Act on healthcare benefits considering the following classifications: saving life and curative, saving life and improving outcomes, preventing the premature death, improving HRQoL without life prolongation; thus no “innovativeness” per se

^ad^Public health interest (interêt santé publique; ISP) is incorporated into the SMR evaluation. ISP considers 3 things: whether the drug contributes to a notable improvement in population health; whether it responds to an identified public health need (e.g. ministerial plans); and whether it allows resources to be reallocated to improve population health

^ae^However, manufacturer dossiers need to include information on the expected number of patients and patient groups for which an added benefit exists as well as costs for the public health system (statutory health insurance)

^af^The following principles are considered: human dignity, need/solidarity, cost-efficiency, societal view

^ag^Factors include cost-effectiveness, clinical need, broad priorities for the NHS, effective use of resources and encouragement of innovation, and any other guidance issued by the Secretary of State

^ah^Regulated by law: the Act on healthcare benefits considering: impact on public health in terms of priorities for public health set; impact on prevalence, incidence—qualitative assessment rather than quantitative

^ai^Only potentially as part of economic evaluations

^aj^Productivity loss due to incapacity as part of the cost side, productivity loss due to mortality as part of the benefit side (no unpaid work, e.g. housework)

^ak^Indirect costs can be taken into account in a separate analysis

^al^No social perspective obligatory by law; may be provided but problematic to use for recommendation/decision

^am^Already implemented but analysis conducted separately by the distinct CEESP. The health economic evaluation does not impact the reimbursement decision

^an^CBA is not standard practice in the evaluation but, rather, can be initiated if no agreement is reached between sickness funds and manufacturer on the price premium or if the manufacturer does not agree with the decision of the G-BA regarding premium pricing (added benefit)

^ao^ASMR V drugs should be listed only if they reduce costs (lower price than comparators or induce cost savings)

^ap^The commission will also make a statement if a drug shall be used as first choice or only if other existing therapeutics are not effective in a patient

^aq^In the form of the new IPT—Informes de Posicionamiento Terapéutico/Therapeutic Positioning reports.

^ar^Sub-groups are examined as part of benefit assessment but in order to guide pricing, not reimbursement eligibility. If a drug has an added benefit for some groups but not for others, a so-called “mixed price” is set that reflects both its added benefit for some patients and lack thereof for others

^as^The assessment in France is purely ‘scientific’ i.e. focuses on the absolute and comparative merits of the new therapy and its placement in the therapeutic strategy

^at^NICE principles include fair distribution of health resources, actively targeting inequalities (SoVJ); equality, non-discrimination and autonomy

^au^Also indirectly through a seat for an ethicist in the Committee

^av^No clear order between “need and solidarity” and cost-efficiency. In the entire health system a more complete ordering is seen where human dignity takes precedence over the principles of need and solidarity, which takes precedence over cost-efficiency

^aw^Not all regions have either HTA agencies or regional committees for drug assessment. However, at regional level drug assessment is limited to prioritizing (or not) its use by means of guidelines or protocols together with some type of incentives to promote savings

^ax^For therapeutic benefit, other designs such as non-randomised or observational studies might be accepted in exceptional cases if properly justified, e.g. in the case that RCTs are not possible to be conducted, if there is a strong preference for a specific therapeutic alternative on behalf of doctors or patients, if other study designs can provide sufficiently robust data, etc

#### Evaluation principles and their relevance to priority setting

In France, the assessment of the product’s medical benefit or medical service rendered (Service Médical Rendu, SMR), and improvement of medical benefit (Amélioration du Service Médical Rendu, ASMR), determine a new drug’s reimbursement and pricing respectively. As of October 2013, economic criteria have been introduced with the Commission for Economic Evaluation and Public Health (CEESP) evaluating the cost-effectiveness (without a cost-effectiveness threshold in place) of products assessed to have an ASMR I, II or III that are likely to impact social health insurance expenditures significantly (total budget impact greater than EUR 20 million); results are used by the Economic Committee for Health Products (CEPS) in its price negotiations with manufacturers [29]. Nevertheless, and under this current framework, these economic evaluations do not have the same impact on price negotiation as does the ASMR, which is linked directly to pricing. Instead, the role of economic evaluations is consultative in this process.

In Germany, the new Act to Reorganize the Pharmaceuticals Market in the Statutory Health Insurance (SHI) System [Gesetz zur Neuordnung des Arzneimittelmarktes in der gesetzlichen Krankenversicherung (AMNOG)] came into effect on 1 January 2011. Since then, all newly introduced drugs are subject to early benefit assessment. Pharmaceutical manufacturers have to submit a benefit dossier for evaluation by the IQWiG. A final decision is made by the Federal Joint Committee (Gemeinsamer Bundesausschuss, G-BA). Benefit for new drugs encompasses the “patient-relevant therapeutic effect, specifically regarding the amelioration of health status, the reduction of disease duration, the extension of survival, the decrease in side effects or the improvement of quality of life” [30]. Importantly, all new drugs are reimbursed upon marketing authorisation, with benefit assessment mainly determining price rather than reimbursement status.

In Sweden, a prioritisation framework with three explicit factors for the allocation of resources is used: (1) human dignity; (2) need and solidarity; and (3) cost-efficiency [31–34]. However, in the specific legislation for the pharmaceutical reimbursement system, human value is generally seen as the overriding criterion with no clear order between the other two [25]. Marginal benefit or utility, according to which a diminishing cost-effectiveness across indications and patient groups is explicitly recognized, could be regarded as a fourth principle, mainly meaning that there are no alternative treatments that are significantly more suitable [31, 35, 36].

In England, the Secretary of State for Health has indicated to NICE a number of factors that should be considered in the evaluation process: (1) the broad balance between benefits and costs (i.e. cost-effectiveness); (2) the degree of clinical need of patients; (3) the broad clinical priorities for the NHS; (4) the effective use of resources and the encouragement of innovation; and (5) any guidance issued by the Secretary of State [37–39]. Decisions are supposed to reflect societal values, underlined by a fundamental social value judgment [40].

The Netherlands focuses on four priority principles when assessing medical technologies: (1) the “necessity” of a drug (severity/burden of disease) [41, 42]; (2) the “effectiveness” of a drug, according to the principles of evidence-based medicine (EBM) [42, 43]; (3) the “cost-effectiveness” of a drug [44]; and (4) “feasibility”, i.e. how feasible and sustainable it is to include the intervention or care provision in the benefits package [45, 46].

In Italy, reimbursement of pharmaceuticals at the central level is evaluated by AIFA’s Pricing and Reimbursement Committee (CPR), which sets prices and reimbursement conditions for drugs with a marketing authorisation based on evidence of the following factors: the product’s therapeutic value (cost/efficacy analysis) and safety (pharmacovigilance), the degree of therapeutic innovation, internal market forecasts (number of potential patients and expected sales), the price of similar products within the same or similar therapeutic category and product prices in other European Union Member States [25]. In autonomous regions, pricing and reimbursement of new drugs does not require—except for very innovative drugs—epidemiologic or economic evaluation studies nor assessment of cost impact from the adoption of new drugs, as in other countries [25, 47].

An HTA in Poland is considered complete if it contains (1) a clinical effectiveness analysis; (2) an economic analysis; and (3) a healthcare system impact analysis. No studies were available from the systematic review referring to  the evidence assessed or the different parameters considered by AOTMiT in Poland [48].

Finally, in Spain different regions apply a range of different assessment requirements, but in general four main evidence parameters are considered: (1) the severity of the disease; (2) the therapeutic value and efficacy of the product; (3) the price of the product; and (4) the budget impact for the Spanish National Health System. The assessment is usually a classification or a cost-consequences analysis that does not take into account the long-term effects of a therapy or the possible need of specialized care utilization. Patient well-being and quality of life are also considered [49].

#### Evaluation criteria taken into account in HTAs

##### Burden of disease

In France, both the severity and the existence of alternative treatments act as formal criteria, thus essentially defining the concept of ‘need’ [41]. Severity is considered as part of the SMR, taking into account symptoms, possible consequences, including physical or cognitive handicap, and disease progression in terms of mortality and morbidity [25]. The existence of alternatives is scored against a binary scale (yes vs. no) [50, 51].

In Germany, severity is considered as part of added (clinical) benefit assessment. The clinical assessment is based on “patient-relevant” outcomes, mainly relating to how the patient survives, functions or feels, essentially accounting for the dimensions of mortality, morbidity and HRQoL [52].

In Sweden, severity of the condition and the availability of treatments reflected through marginal benefit/utility as a sub-principle appear to be two of the primary criteria for priority-setting, with more severe indications being explicitly prioritized via greater willingness to pay (WTP) [31, 35, 36, 41].

In England, the degree of unmet clinical need is a formal criterion taken into account, being reflected by the availability of alternative treatments [41, 53]. NICE acknowledges that rarity plays a key role in the assessment of orphans and NICE’s Citizens’ Council has stated that society would be willing to pay more for rare and serious diseases [54]. The severity of the disease is taken into account mainly through the special status of life-extending medicines for patients with short-life expectancy as reflected through the issuing of supplementary advice of life-extending end-of-life (EOL) treatments by NICE [53, 55].

Severity of disease, availability of treatments, and prevalence of the disease are generally considered across the remaining countries, either explicitly or implicitly, although not always as mandatory requirements by law but just as good HTA practices (e.g. as in Poland for the case of treatments availability) [25].

##### Therapeutic impact and safety

Clinical evidence relating to therapeutic efficacy and safety acts as the most important formal criterion of the evaluation process in France [56]. The product’s SMR relates to the actual clinical benefit, responding to the question of whether the drug is of sufficient interest to be covered by social health insurance. It takes into consideration the following criteria: (1) the seriousness of the condition; (2) the treatment’s efficacy; (3) side effects; (4) the product’s position within the therapeutic strategy given other available therapies; and (5) any public health impact [25, 27].

Similarly to France, in Germany all clinically relevant outcomes are considered and final clinically meaningful outcomes (e.g. increase in overall survival, reduction of disease duration, improvement in HRQoL) are preferred over surrogate and composite endpoints [27, 28, 52, 57, 58]. HRQoL endpoints are considered if measured using validated instruments suited for application in clinical trials [25, 30]. With regards to uncertainty, IQWiG ranks the results of a study according to “high certainty” (randomized study with low bias risk), “moderate” (randomized study with high bias risk), and “low certainty” (non-randomized comparative study). The complete evidence base is then assessed and a conclusion is reached on the probability of the (added) benefit and harm, graded according to major added benefit, considerable added benefit, and minor added benefit. Three additional categories are recognized: non-quantifiable added benefit, no added benefit, and lesser benefit [25, 52].

All types of clinically relevant outcomes are accepted in Sweden, including final outcomes, surrogate endpoints, and composite endpoints, with generic QoL endpoints being preferred over disease-specific endpoints [25, 57]. Generally, all effects of a person’s health and QoL are supposed to be considered as part of the assessment stage, including treatment efficacy and side effects [35, 36, 56].

In England, data on all clinically relevant outcomes are accepted with final clinical outcomes (e.g. life years gained) and patient HRQoL being preferred over intermediate outcomes (e.g. events avoided) or surrogate endpoints and physiological measures (e.g. blood glucose levels) [57, 59–61]; particular outcomes of interest include mortality and morbidity. Safety is addressed mainly through the observation of adverse events [53]. Uncertainty is addressed explicitly through quality of evidence, implicitly through preference for RCTs, and indirectly by rejecting a submission if evidence is not scientifically robust.

Italy, the Netherlands, Poland and Spain include surrogate and composite endpoints in the analysis, in addition to disease-specific quality of life endpoints. Therapeutic value is the most critical criterion for reimbursement in the Netherlands, as part of which patient preference data and user friendliness may also be considered [43].

All countries take into consideration safety data to reflect clinical harm, mainly in the form of the incidence and severity of adverse events.

##### Innovation level

In the French setting, clinical novelty is considered by definition through the product’s ASMR relating to its relative added clinical value, which informs pricing negotiations [25]. Additional innovation characteristics relating to the nature of the treatment (e.g. differentiating between symptomatic, preventive and curative) are also considered, but as a second line of criteria [25, 56, 61, 62].

In Germany, clinical novelty is considered implicitly as part of the consideration of added therapeutic benefit for premium pricing. Ease of use and comfort (if relevant for morbidity or side effects) can be reflected indirectly through treatment satisfaction for patients, which can be considered as an additional aspect but not as an explicit factor, similarly to the nature of the treatment/technology [63].

In Sweden, innovation characteristics relating to the added therapeutic benefit (only if it can be captured in the CE analysis), as well as ease of use and comfort are included in the assessment process [25, 41, 56, 61].

As reflected through NICE’s operational principles, the encouragement of innovation is an important consideration in England. By definition, the incremental therapeutic benefit as well as the innovative nature of the technology are formally taken into account as part of the product’s incremental cost effectiveness ratio (ICER) [53].

Among the remaining countries, clinical novelty is essentially considered in all countries; ease of use and comfort might only be considered implicitly and informally if at all, whereas there are mixed approaches in terms of a treatment’s technology nature.

##### Socioeconomic impact

In terms of socioeconomic parameters, in France ‘expected’ public health benefit acts as another explicit dimension via an indicator known as public health interest (“Intérêt de Santé Publique”, ISP), which is assessed and scored separately by a distinct committee as part of the SMR evaluation but not used often [25, 41, 62, 64].

In Germany, public health benefit is not explicitly considered but only partially reflected through the requirement from manufacturers to submit information on the expected number of patients and patient groups for which an added benefit exists, as well as costs for the public health system (statutory health insurance) [25, 63]. All direct costs have to be considered, including both medical and non-medical (when applicable), whereas indirect costs are not a primary consideration but can be evaluated separately if they are substantial, with productivity losses due to incapacity being included only on the cost side [65]. In turn, productivity losses due to mortality are considered in the outcome only on the benefit side (to avoid double counting). Budget impact analysis (BIA) is mandatory and should include any one-off investments or start-up costs required in order to implement a new technology, with methodology and sources clearly outlined [27, 65].

Among the other study countries, any public health impact of the drug is usually considered, but not necessarily in an explicit manner, whereas social productivity might be reflected through the incorporation of indirect costs, either explicitly or implicitly [25]. In England for example, although productivity costs should be excluded, cost of time spent on informal caregiving can be presented separately if this care might otherwise have been provided by the NHS or personal social services (PSS) [66].

##### Efficiency

In France, up until now cost-effectiveness was not acknowledged as an explicit or mandatory criterion, but BIA, while not mandatory, is highly recommended [25]. Although the expert committee had been reluctant to use cost-effectiveness criteria in the evaluation process [56, 67], following a bylaw passed in 2012 (which took effect in 2013) the role of economic evidence was strengthened [51]. The CEESP gives an opinion on the efficiency of the drug based on the ASMR of alternative treatments.

In Germany, economic analysis [cost-benefit-analysis (CBA)] is not standard practice in the evaluation, but, rather, is optional and can be initiated if no agreement is reached between sickness funds and the manufacturer on the price premium, or if the manufacturer does not agree with the decision of the G-BA regarding premium pricing (added benefit); instead, BIA is mandatory (Advance-HTA, 2016). ‘Cost-effectiveness’ acts as one of the most important formal evaluation criteria in Sweden. Parameters having a socioeconomic impact, such as avoiding doctor visits or surgery, productivity impact, and, in general, savings on direct and indirect costs are also considered [35].

As already reflected through NICE’s working principles, the relative balance between costs and benefits (i.e. value-for-money), and the effective use of resources should be taken into account in England (e.g. through the explicit cost-effectiveness criterion) [37]. Some studies also suggest that the impact of cost to the NHS in combination with budget constraints (budget impact considerations) are taken into account alongside the other clinical and cost-effectiveness evidence [39, 67–70].

In the assessment process by ZIN, the cost-effectiveness criterion follows that of the therapeutic value and the cost consequences analysis. Cost-effectiveness is only considered for drugs with added therapeutic value, which are either part of a cluster and are reimbursed at most at the cluster’s reference price, or are not reimbursed in the absence of possible clustering [43, 71]. The Netherlands usually performs its own BIA, although voluntary submission from the manufacturer is also an option [43, 67].

All other study countries evaluate the efficiency of new drugs through cost-effectiveness evaluation and BIA, but this is not always mandatory or an explicit criterion in value assessment and pricing/reimbursement negotiations.

##### Other types of evidence

Additional explicit parameters considered in France include the technology’s place in the therapeutic strategy, mainly in relation to other available treatments (i.e. first-line treatment vs. second-line treatment etc.), and the technology’s conditions of use [25, 50, 51].

Germany is the only country that does not apply any conditions of use in regards to specific sub-populations, in principle reimbursing drugs across the whole indication spectrum as listed on the marketing authorisation [25]. Nevertheless, recent IQWiG appraisals increasingly focus on providing value assessments at sub-population level.

As reflected through the ethical prioritisation framework used by the Swedish TLV, the ethical considerations of human dignity, need and solidarity act as principles for the evaluations.

Besides the notion of clinical need as reflected through NICE’s principles, other equity considerations include the ‘need to distribute health resources in the fairest way within society as a whole’ and the aim of ‘actively targeting inequalities’, both of which are explicitly mentioned by NICE as principles of social value judgements [37]. Equality, non-discrimination, and autonomy are other explicit ethical considerations [41].

The Netherlands also takes into consideration explicitly ethical criteria based on egalitarian principles, such as solidarity and affordability of the technology by individual patients [25, 33, 41].

In terms of the remaining countries, conditions for use may be placed in Italy, Poland and Spain, the therapy’s place in therapeutic strategy considerations exist for Italy and Spain, whereas ethical considerations are evident in Italy and Poland (implicitly or indirectly). However, the use of any additional explicit parameters may not be transparent in these settings.

#### Synthesizing the evidence and taking into account all factors: weights

It is not clear how all the factors discussed so far interact with one another, what their relative importance is and what the trade-offs are that HTA agencies are prepared to make between them when arriving at recommendations [70, 72]. For example, in France the weights of the assessment parameters considered and the appraisal process overall do not seem to be clear or transparent [56], although the evidence that informs this judgment is dated and may be contestable. In Spain, the assessment takes into account mainly safety, efficacy, effectiveness, and accessibility and it does not consider explicitly efficiency and opportunity cost; still the way this is undertaken and the weights of different criteria remain unknown [73]. All countries consider a number of different data sources for the assessment process, with randomised controlled trials (RCTs) usually being the most preferred source for clinical data.

### HTA methods and techniques applied

Assuming the existence of an additional benefit (or lesser harm) compared to existing treatment options, all countries with the exception of France and Germany are adopting some type of economic evaluation, mainly cost utility analysis (CUA) or cost-effectiveness analysis (CEA), as the analytical tool to arrive at value-for-money recommendations aiming at improving effiiency in resource allocation; both France and Germany used to apply a comparative assessment of clinical benefit as the sole methodology, with economic evaluation progressively becoming more important in France as of 2013 but in the context of the existing method of assessment. A summary of analytical methods and techniques applied as part of HTA and their details is presented in Table 3.

**Table 3:** HTA methods and techniques applied

	France (HAS/CEESP^a^)	Germany (IQWiG)	Sweden (TLV)	England (NICE)	Italy (AIFA)	Netherlands (ZIN)	Poland (AOTMiT)	Spain (RedETS/ISCIII or ICP)
**Analysis method**								
Methods	Comparative efficacy/effectiveness (also CEA, CUA)	CBA but also CUA and CEA (not standard practice)	CUA (also CEA, CBA)	CUA (also CEA, CMA)	CMA, CEA, CUA, CBA^b^	CEA, CUA, no CMA	Cost-consequences analysis, CEA or CUA—obligatory, CMA (if applicable)	Comparative efficacy/effectiveness, CMA, CEA, CUA, CBA^c^
Preferred outcome measure	Final outcome, life years (QALY, if CUA; life years, if CEA)	Patient relevant outcome (can be multidimensional)—efficiency frontier	QALY (WTP, if CBA)	QALY (cost per life year gained, if CEA)	Final outcome, life years (QALY, if CUA or CEA; life years, if CEA)	Effectiveness by intention-to-treat principle, and expressed in natural units—preferably LYG or QALY	QALY or LYG	QALY in CUA
Utility scores elicitation technique	EQ-5D and HUI3, from general French population	Utility scores from patients, direct (e.g. TTO, SG), indirect	Utility scores from patients, direct (e.g. TTO, SG), indirect (EQ-5D)	Utility scores from general English population, direct (e.g. TTO, SG), indirect (EQ-5D), systematic review	Both direct and indirect (EQ-5D) elicitation techniques	Either direct (TTO, SG, VAS), or indirect (EQ-5D); selection should be justified	Direct or indirect utility scores^d^	Utility scores from general Spanish population, direct (e.g. TTO, SG), indirect (EQ-5D)^e^
Comparator	Usually ‘best standard of care’ but can be more than one^f^	Usually ‘best standard of care’ but can be more than one^g^	Usually ‘best standard of care’ but can be more than one^h^	Usually ‘best standard of care’ but can be more than one^I^	Usually ‘best standard of care’ but can be more than one^j^	Treatment in clinical guidelines of GPs; if not available, most prevalent treatment	‘Best standard of care’ which is reimbursed in Poland^k^	Best standard of care, usual care and/or more cost-effective alternative
Perspective	Widest possible to include all health system stakeholders^l^	Usually statutory health insurant^m^	Societal	Cost payer (NHS) or societal if justified	Italian National Health Service^n^	Societal (report indirect costs separately)	The public payer’s perspective, public payer and patient (by law)	Cost payer (NHS) and societal (rarely used), and they should be presented separately
Subgroup analysis	Yes (when justified)	Yes	Yes	Yes	Yes	Yes	Yes (if needed, but decreases validity)	Yes
**Clinical evidence**								
Preferred study design	Head-to-head RCTs; other designs accepted if no RCTs available	Head-to-head RCTs; other designs accepted in the absence of RCTs	Head-to-head RCTs; other designs accepted if no RCTs available	Head-to-head RCTs; other designs accepted if no RCTs available	Head-to-head RCTs; other designs accepted if no RCTs available	Head-to-head RCTs	Head-to-head RCTs; other designs accepted if no RCTs available	Head-to-head RCTs; other designs accepted if no RCTs available
Systematic literature reviews for collecting evidence required/conducted by regulator	Yes, guidelines provided/yes, in French	Yes/no	Not mandatory	Yes/yes	Yes/yes	Yes/yes	Yes	Not always^o^
Meta-analysis for pooling evidence	Not specified	Not specified for new drugs	Not specified	Yes	Yes	Yes, encouraged	Yes	No^p^
Data extrapolation	Qualitative only, in absence of effectiveness data form RCTs	No	Quantitative, both in absence of RCT effectiveness data and in absence of long-term effects	Qualitative and quantitative, both in absence of RCT effectiveness data and in absence of long-term effects	Quantitative Qualitative in absence of RCT effectiveness data	Qualitative, in the absence of RCTs and in absence of long-term effects	Possible if needed but not recommended	Quantitative, in the absence of effectiveness data
**Resources/costs**								
Types	Direct medical, direct non-medical, indirect (both for patient and carer)	Depending on perspective: direct medical, informal costs, productivity loss (as costs)	Direct medical, direct non-medical, indirect (both for patient and carer)	Direct medical, social services	Direct costs only; indirect costs can be taken into account in a separate analysis	Both direct and indirect costs inside and outside the healthcare system	Direct medical costs, direct non-medical costs	Direct and indirect costs (on rare occasions), costs of labour production losses or lost time, informal care costs
Data source/unit costs	Direct: PMSI (Programme de Médicalisation des Systèmes d’Information) Indirect: human capital costing, friction costing	Statutory health insurance, further considerations depending on perspective chosen	Drugs: pharmacy prices Indirect: human capital costing	Official DoH listing	Variety of sources^q^	Reference prices list should be used	Variety of sources^r^	Official publications, accounts of health care centres, and the fees applied to NHS service provision contracts
**Discounting**								
Costs	4% (up to 30 years) and 2% after	3%	3%	3.5%	Not available (update in progress)	4%	5%	3%
Outcomes	4% (up to 30 years) and 2% after	3%	3%	3.5%	Not available (update in progress)	Under review—will probably be set at same level as costs discounting	3.5%	3%
Sensitivity analysis	0%, 3% (6% max)	0–5%	0–5%	0–6%	Not available (update in progress)	Not obligatory	5 and 0% for costs and outcomes 0% for outcomes 5% for costs^s^	0–5%
**Time horizon**								
Time horizon	Long enough so that all treatment outcomes can be included	At least the average (clinical) study duration; longer for chronic conditions, especially if lifetime gains are expected; same horizon for costs and benefits	Time needed to cover all main outcomes and costs	Long enough to reflect any differences on outcomes and costs between technologies compared	Duration of the trial is considered^t^	Primarily based on duration of RCTs^u^	Long enough to allow proper assessment of differences in health outcomes and costs between the assessed health technology and the comparators	Should capture all relevant differences in costs and in the effects of health treatments and resources^v^
**Thresholds**								
Thresholds	No threshold (only eligibility threshold to conduct economic evaluation)	Efficiency frontier (Institute’s own approach)	No official threshold; 50% likelihood of approval for ICER between €79,400 and €111,700	£20,000–£30,000 per QALY; Empirical: £12,936 per QALY	No threshold in use	No official threshold	3 × GDP per capita for ICUR(QALY) or ICER(LYG)	Unofficial: €21,000–€24,000/QALY (recently provided by SESCS^w^ to the Spanish MoH)

*Source* The authors (based on literature review findings and expert consultation)

* CEA* Cost-effectiveness analysis,* CUA* cost utility analysis,* CMA* cost minimization analysis,* QALY* quality adjusted life year,* LYG* life year gained,* TTO* time trade off,* SG* standard gamble

^a^In France, economic evaluations are undertaken only for selected drugs with expected significant budget impact

^b^A template for the submission of the pricing and reimbursement (P&R) dossier to AIFA is in progress

^c^For the case of drugs at central level carried out by ICP, comparative efficacy/effectiveness is taken into account. The ICP receives the so called “Informe de Posicionamiento Terapéutico” (Therapeutic Positioning report), a therapeutic assessment conducted by the Spanish Medicines Agency (Agencia Española del Medicamento) based on which confidential discussions around the appraisal of the drugs takes place but which does not take into consideration cost-effectiveness. Economic evaluations are mainly taking place for the case of non-drug technologies under the scope of RedETS

^d^It is recommended to use indirect methods for preferences measurement—validated questionnaires in Polish. While measuring preferences with the EQ-5D questionnaire, it is advised to use the Polish utility standard set obtained by means of TTO

^e^Surveys or previously validated HRQOL patient surveys

^f^Including most cost-effective, least expensive, most routinely used, and newest

^g^Including most cost-effective, least expensive, and most routinely used. If the efficiency frontier approach is used as part of CBA, then “all relevant comparators within the given indication field” must be considered

^h^Including most cost-effective, least expensive, and most routinely used

^I^Including most cost-effective, least expensive, most and routinely used

^j^Including most cost-effective, and most routinely used

^k^These might include (1) most frequently used; (2) cheapest; (3) most effective; and (4) compliant to the practical guidelines

^l^Needs justification (especially if societal)

^m^Also community of statutorily insured, perspective of individual insurers, or the societal perspectives are possible

^n^Societal perspective is not mandatory, but can be provided in separate analysis

^o^For non-drugs under RedETS, a systematic literature review is always conducted

^p^For non-drugs under RedETS, a meta-analysis may be conducted

^q^Prices available in the Official Journal of the Italian Republic (Gazzetta Ufficiale), accounts of health care centres, the fees applied to NHS service, scientific literature/ad hoc studies

^r^Including (1) list of standard costs, (2) formerly published research, (3) local scales of charges, (4) direct calculation

^s^It is currently under revision (AOTMiT HTA Guidelines updating process) and may change soon

^t^Additional long-term evidence collected through monitoring registries

^u^Secondary horizons include any longer needed depending on the context of interest

^v^In some cases, the time horizon will have to be extended to the individual’s entire lifespan

^w^Servicio de Evaluación y Planificación, Islas Canarias

#### Analytical methods

In Sweden and England the preferred type of economic evaluation is CUA with cost per QALY gained being the favoured health outcome measure, but CEA being also accepted if there is supporting evidence to do so (as in the case that the use of QALY for a particular case seems inappropriate) [27, 28, 37, 38, 60, 74–77]. In Sweden, CBA with WTP as an outcome measure can also be applied.

In France, up until now comparative assessment of clinical benefit incorporating final endpoints as an outcome measure acted as the preferred evaluation procedure. However, economic analysis of selected drugs with expected significant budget impact is continuously being considered more formally, especially if its choice is justified and any methodological challenges (especially associated with the estimation of QALYs) are successfully addressed [27, 28, 41, 50, 51, 58]. The choice between CEA and CUA depends on the nature of the expected health effects (if there is expected significant impact on HRQoL then CUA is used, otherwise CEA).

In Germany, economic evaluations are performed within therapeutic areas and not across indications, thus, an efficiency frontier approach of CBA using patient relevant outcomes is the preferred combination of analysis method and outcome measure [22, 27, 28, 58, 65]. Since the introduction of the AMNOG, economic evaluations are supposed to be conducted for cases when price negotiations fail after the early benefit assessment and the verdict is challenged by the technology supplier or the statutory health insurer [65]. However, no such analysis has been submitted so far and seems unlikely to ever happen because the CBA would have to be re-evaluated by IQWiG, which would hardly bring any better results [25].

In the Netherlands and Italy, the preferred type of economic evaluation is CUA if the improvement in quality of life forms an important effect of the drug being assessed, or if this is not the case, a CEA [78, 79]. In Spain, any of the four methods of analysis may be used (CMA, CEA, CUA or CBA).

#### Types of clinical evidence considered

In relation to clinical evidence, all countries acknowledge that randomised controlled head-to-head clinical trials are the most reliable and preferred source of treatment effects (i.e. outcomes), with data from less-rigorous study designs being accepted in most study countries (England, France, Germany, Sweden, Poland, Spain, Italy), e.g. when direct RCTs for the comparators of interest are not available [28, 53, 61].

Most agencies require systematic literature reviews to be submitted by manufacturers as a source of data collection, and carry out their own reviews. A meta-analysis of key-clinical outcomes is recommended for pooling the results together given the homogeneity of the evidence in England, Italy, Netherlands and Poland [28, 53].

If evidence on effectiveness is not available through clinical trials, France and the Netherlands allow for a qualitative extrapolation based on efficacy data, with Spain conducting quantitative extrapolation, and Sweden, England, Italy and Poland applying both qualitative and quantitative modelling. In Sweden, England and Netherlands, short-term clinical data are extrapolated also if data on long-term effects are absent.

#### Resources/cost evidence

In terms of resources used, in addition to direct medical costs, France and Sweden consider all relevant costs, including direct non-medical and indirect costs, both for patients and carers [27, 28]; however, only direct costs are considered in the reference case analysis and incorporated in the ICER in the case of France [50]. Germany also takes into account informal costs and productivity gains separately as a type of benefit, whereas England additionally considers cost of social services.

Poland incorporates direct medical costs and direct non-medical costs. In the Netherlands, the Health Care Insurance Board’s “Manual for cost research” applies for the identification, measurement and valuation of costs; pharmacoeconomic evaluations need to include both direct and indirect costs inside and outside the healthcare system [78]. In Italy, it is recommended to include direct costs; indirect costs can be taken into account in a separate analysis [25]. Spain incorporates both direct and indirect costs (the latter on rare occasions), as well as costs of labour production losses or lost time and informal care costs, in the analysis [25, 58]. Finally, all countries recommend the application of country-specific unit costs [28].

#### Discounting and time horizon

In all study countries, both costs and benefits are discounted [27, 58, 61, 74], and uncertainty arising due to variability in model assumptions is investigated, usually in the form of a sensitivity analysis. In Italy, information on discounting is not available at the moment due to an update in progress by AIFA [25]. In terms of a suitable time horizon, none of the countries use an explicit time frame but, instead, they adopt a period that is long enough to reflect all the associated outcomes and costs of the treatments being evaluated, including the natural course of the disease [27, 80].

#### Acceptable ‘value for money’ thresholds

No explicit, transparent, or clearly defined cost-effectiveness thresholds exist in any of the countries except for England, Poland, and an academic proposal for Spain.

In line with the World Health Organization (WHO) suggestions of two to three times the gross domestic product (GDP) per capita, a three times GDP per capita threshold has been implemented in Poland. Generally, a drug is deemed cost-effective by AOTMiT if cost per QALY estimates are less than three times the GDP per capita (but smaller than 70,000 PLN per QALY/LYG) [25,81].

In Spain, a €21,000–€24,000 per QALY threshold was recently provided by Servicio de Evaluación y Planificación Canarias (SESCS) to the Ministry of Health; however, this might not be actively adopted in practice [25].

In England, although evidence suggests the existence of a threshold ranging somewhere between £20,000 and £30,000 [44, 59, 75, 82], it is evident that such a threshold range might not be strictly applied in practice, with some products having a cost per QALY below these ranges receiving negative coverage recommendations, and other products above these ranges ending up with positive recommendations [60, 83, 84]. Indeed, several studies point towards the existence of a threshold range based on which additional evidence on several factors is required for the recommendation of technologies with an ICER of above £20,000, and even stronger evidence of benefit in combination with explicit reasoning required for the coverage of technologies with an ICER above £30,000 [38, 39, 44, 53, 56, 85]. However, a more recent study using data on primary care trust spending and disease-specific mortality estimated an empirical based “central” threshold of £12,936 per QALY, with a probability of 0.89 of less than £20,000 and a probability of 0.97 to be less than £30,000 [86].

In Germany, the efficiency frontier approach is used to determine an acceptable “value for money”, even though this is not involved in the process of the initial rebate negotiations. In Sweden, recent evidence suggested that the likelihood of approval is estimated to be 50% for an ICER between €79,400 and €111,700, for non-severe and severe diseases respectively [87].

In the Netherlands, there is no formal threshold in place but there have been some attempts to define one. The €20,000 per life-year gained (LYG) threshold used in the 1990s to label patients with high cholesterol levels eligible for treatment with statins has been mentioned in discussions on rationing, but was never used as a formal threshold for cost-effectiveness. The same was the case with a threshold that the Council for Care and Public Health wanted to implement based on criteria such as the GDP per capita, in line WHO recommendations, which, for the Netherlands, would translate into €80,000/QALY [71]. The Council also suggested that the cost per QALY may be higher for very severe conditions (a tentative maximum of €80,000) than for mild conditions (where a threshold of €20,000 or less may apply) [46], but none of the above was ever implemented.

### HTA outcomes and implementation

In all countries, assessment and appraisal of outcomes are used mainly as a tool to inform coverage recommendations relating to the reimbursement status of the relevant technologies; all countries use the results to inform pricing decisions directly or indirectly. A summary of the types of HTA outcomes and their implementation in the study countries is presented in Table 4.

**Table 4:** HTA outcomes and implementation

	France (HAS/CEESP)	Germany (IQWiG)	Sweden (TLV)	England (NICE)	Italy (AIFA)	Netherlands (ZIN)	Poland (AOTMiT)	Spain (RedETS/ISCIII or ICP)
Publicly available report	Yes, both in French and English^a^	Yes	Yes (summary report with some details on cost-effectiveness)	Yes	Yes, in the Official Journal of the Italian Republic (Gazetta Ufficiale)	Yes	Yes (in Polish on the AOTMiT website), but confidential information is publicly unavailable	No for drugs^b^
**Policy implication**								
Reimbursement	Yes, through SMR^c^	Indirectly	Yes	Yes	Yes	Yes	Yes	Yes
Pricing	Yes, through ASMR^d^	Indirectly	Yes	Only indirectly as it has an impact on product’s ICER	Yes	Yes, except certain expensive medicines^e^	Yes, if reimbursement decision is positive	Yes
Access restrictions	Yes, various restrictions in place^f^	Existence of managed entry agreements but details not publicly available	Yes, restrictions for specific subpopulations, temporary decisions and risk sharing agreements	Yes, major and minor restrictions as well as performance based agreements	Yes, various managed entry agreements^g^	Yes, system of CED	Yes, including major and minor^h^	Yes
Dissemination	Publicly available online	Dossier assessment, reports, rapid reports, addendums and patient information websites	Informational material distributed to the major stakeholders, decisions published online	Publicly available online	Monthly AIFA publication of price lists of reimbursed products. Annual publication of data on pharmaceutical expenditure and consumption (Rapporto Osmed)	Online for general public and distributed to stakeholders	Publication online	No for drugs^I^
Implementation	Prescription guidelines, drug formularies and positive list	Prescription advice issued by G-BA based on therapeutic assessment (“Therapiehinweise”)	Drug formularies	Prescription guidelines, drug formularies	A product can be assigned to Class A, H or C^j^	Positive list; in case of therapeutic equivalent, the drug is either not accepted for public reimbursement or subject to a reference pricing system	Different reimbursement lists categories^k^	Inclusion in the national reimbursement list
Appeal	Yes^l^	Yes, through arbitration board^m^	Yes	Yes	Companies can appeal to Court but there is no specific appeal procedure	Yes	No	Yes
Revision	Yes, every 5 years or sooner if decision from HAS or request from the MoH	Yes, at least one year after benefit assessment^n^	Yes	Yes	Yes^o^	Yes, but not on a regular basis^p^	Yes, 2 years after first assessment, 3 year after 2nd, 5 years after 3rd assessment	Yes

* Source* The authors (based on literature review findings and expert consultation)

* CED* Coverage with evidence development

^a^Economic evaluation reports are available but some parameters are deleted in the public version (elements related to medicines costs mainly)

^b^For non-drug technologies under RedETS usually yes, in the form of bulletins and web pages of HTA agencies

^c^The level of SMR determines if a drug shall be reimbursed and, if yes, at which level (low 15%, moderate 30%, high 65%)

^d^The level of ASMR is used for pricing negotiations with manufacturers

^e^A bureau of the government negotiates rebates with the industry on a case-by-case approach for certain expensive medicines (actual price is ‘secret’ but hospitals can ask for an add-on)

^f^Including recommendation to only reimburse this medicine in second intention, restrictions to specific sub-populations, Financial risk-sharing (price–volume agreements and budget caps)

^g^Such as price–volume agreements, cost-sharing, budget cap, monitoring registries, payment by results, risk-sharing, therapeutic plans, and “AIFA notes”

^h^‘Major’ include restricted to specific subpopulations (monitoring of use); ‘minor’ include requiring a lower price so called Risk Sharing Schemes (cost sharing in practice)

^I^RedETS reports for non-drugs become publicly available

^j^Class A refers to products reimbursed by the NHS. Class H refers to products for hospital use. Class C refers to non-reimbursed products

^k^Pharmacy drugs (Rx drugs; 30 or 50% patient co-payment, lump sum, no co-payment); drug programmes (selected diseases and patients; free); chemotherapy drugs (hospital settings; free); drugs reimbursed in off-label indications

^l^Manufacturers can appeal decisions made by both commissions. They are then called for an audition to explain their position

^m^Manufacturers have the right to commission CBA if they do not agree with the established added benefit

^n^In some cases decisions are time-limited; revision takes place once the term is over

^o^The negotiation process leads to a 2 year confidential, renewable contract between AIFA and the manufacturer

^p^In practice, providers that have no adequate reimbursement due to a new innovation will ask the Dutch healthcare authority for a revision of reimbursement. The agency then investigates if a revision is reasonable and what the new reimbursement should be

#### Timing and public availability

Generally, the time needed for the evaluation of a health technology to be completed differs from country to country. However, in line with the EU Transparency Directive, all countries must have reached a decision on pricing and reimbursement within 180 days post marketing authorisation [56]. In all countries, the final decision report is publicly available, usually through the HTA agency’s website [12, 56], and the policy implication of the evaluation outcome relates to the pricing and reimbursement status of the technology: reimbursement (list), no reimbursement (do not list), or conditional reimbursement (list with restrictions) [56, 68].

#### Policy implications

In France and Sweden, only drugs with additional therapeutic value can “obtain a higher reimbursement basis” [56]; in France, by assessing the evidence of the product’s medical benefit or medical service rendered (SMR), the improvement in medical benefit and added therapeutic benefit (ASMR) are derived, which determine the reimbursement status and influence the price level of the product respectively, whereas in Sweden the outcome of the evaluation can also drive the price setting in addition to coverage decisions [35, 36].

In Germany, the outcome of the clinical/economic evaluation will be used mainly to inform the negotiation between sickness funds and manufacturer on the price premium. In England, reimbursement status has no direct effects on price, but price indirectly affects the reimbursement status of the drug as it will have an impact on the ICER. In the Netherlands, the positive outcome of an HTA results in the inclusion of the medical technology in the positive list [43]; in terms of the reimbursement decision, if the CEA for a new innovative drug is of high quality, reimbursement will in principle not be denied on the basis of cost-effectiveness, despite potentially relatively high cost-per-QALY values [71]. Finally, in Italy, if a reimbursement status is approved, the pricing is decided simultaneously. If the reimbursement decision is negative, the product will be put on the negative list and the price is determined by the manufacturer (“free pricing”).

#### Access restrictions

All countries apply access restrictions, usually relating to specific indications or specific population sub-groups. France mainly uses financial risk-sharing (price–volume) agreements [56]. Sweden issues temporary decisions for cases when there is insufficient certainty around the (clinical) evidence [56], and risk sharing agreements may take place to speed up the reimbursement process upon the requirement of additional evidence following the review [31], in addition to restricting access for specific sub-populations. In England, major and minor restrictions exist: the former relate to cases where the technology is indicated only for second-line treatment (and beyond), or only for specific sub-populations, and the latter relate to the need for specialist supervision or treatment monitoring [39]; performance based agreements (also known as patient access schemes) also exist, especially in regards to the use of biologics and cancer drugs, according to which a pre-specified clinical (endpoint) condition must be reached at a specific post-assessment time point, i.e. response rules, for the coverage of the technology to continue [88]. The inclusion of expensive cancer drugs which are deemed cost-ineffective in the cancer drugs fund (CDF) is indicative of efforts to enable access to very costly medicines to patients that need them on a selective basis. 

In the Netherlands, the system of coverage with evidence development (CED) for high cost and orphan inpatient drugs was used extensively between 2006 and 2011. Currently, financial-based agreements and performance-based risk sharing agreements are considered as well. In Poland, restrictions could be applied to a positive recommendation, which can be either major, e.g. restricted to specific subpopulations (monitoring of use), or minor, e.g. requiring a lower price (so called Risk Sharing Schemes, but cost sharing in practice) [25]. In Spain, MEAs are concluded at the regional level. Price volume agreements (PVAs) are usually applied to single new products where the negotiated price is conditional on the expected number of units sold.

#### Dissemination and implementation

Most countries employ dissemination procedures in order to support the implementation of their decisions, including prescribing guidelines and national drug formularies [43]. In France, since 2013, there is a public online drug database allowing the general public to access data and documents on marketed drugs [89]. In Germany, IQWiG prepares a variety of dissemination products besides the dossier assessment including technical scientific reports (and rapid reports where no commenting procedures take place), but also public and user-friendly health information and working papers on recent developments in the field, including methodological aspects [52]. The dossier assessment is provided by the G-BA, which can also issue prescribing advice [25]. In Sweden, at least for the review of products that are already on the positive list, informational material in the form of a fact sheet is produced (possibly accompanied by supplementary information taking the form of a PowerPoint presentation and an FAQ sheet), covering the analysis, the appraisal and the conclusion of the evaluation, distributed to the major stakeholders on the date of the decision and about a week before it becomes publicly available online [35, 36]. In England, the NHS is legally obliged to implement NICE guidance and fund the recommended technologies within 3 months of the outcome of the decision [53, 60]. In Poland, since the Reimbursement Act (issued in 2011, effective from 1 January 2012), drugs can be reimbursed under different lists [25]. Pharmacy reimbursement includes prescribed-only medicines available to patients through four main categories of co-payment. Chemotherapy drugs are available in hospital settings free of charge. Other “regimen” programs are available, under which drugs for selected diseases are reimbursed fully to strictly defined patient populations whose eligibility is decided by appropriate clinician committees.

#### Appeal mechanisms and review of decisions

Most countries have appeal mechanisms in place in case of dissent and they all revise their decisions either according to fixed time schedule or on a rolling basis [56, 61]; in France, the drug registration is subject to renewal every five years and a drug may also be subject to post-registration studies. Sweden re-evaluates its old reimbursement list and both Sweden and England may revise technologies once new evidence becomes available. On average, positive recommendations (with or without restrictions) account for approximately  90% of NICE’s appraisals [90].

Although it appears that revisions were taking place systematically after four years for in-patient drugs and on an ad hoc basis for out-patient drugs [42, 56], more recent evidence suggests that, in practice, the process is irregular and providers that have no adequate reimbursement due to a new innovation will ask the Dutch healthcare authority for a revision of reimbursement. The agency then investigates if a revision is reasonable and what the new reimbursement should be [25]. In Italy, the negotiation process leads to a 2-year, confidential, renewable contract between AIFA and the manufacturer [25]; a possible revision is feasible on the grounds of a new product exceeding the original forecast of a company.

## Discussion

In all study countries, HTA agencies have an autonomous function. The evaluation process of medical technologies typically involves an initial assessment of evidence conducted by technical groups, followed by the appraisal of the assessed evidence from an expert committee that is producing reimbursement and coverage recommendation(s) for the final decision body, which can be either the payer (e.g. MoH, HIF), or the HTA agency itself.

In addition to the comparative assessment of clinical benefit, most countries implement a type of economic evaluation (mainly CUA or CEA) as the main analytical method to determine the value of new technologies, with the preferred health gain measure usually being the QALY, or alternative patient-relevant (if not final) outcomes. Both direct preference-based elicitation techniques (e.g. TTO, SG) and indirect multi-attribute classification systems (e.g. EQ-5D and HUI3) are used to elicit utility scores either from patients or the general population. The debate around preferred health gain measures is strong and often contradictory across jurisdictions. For example, while NICE in England favours the use of the QALY, IQWiG in Germany strongly opposes its use on the grounds that it does not reflect patient-level utilities being the ones that actually matter, rather than population-based utilities [25].

The evaluation (assessment and appraisal) outcome is used mainly as an aid to make coverage recommendations in relation to the reimbursement status of medical technologies, but the analysis outcomes are also used to influence pricing decisions as well (although this is done only indirectly in England). Access restrictions for sub-populations or sub-indications, possibly through the application of risk-sharing agreements, have become common practice across many jurisdictions. Information material is often disseminated by the HTA agencies to a range of stakeholder groups; the implementation of agencies’ recommendations is usually taking the form of prescribing guidelines and inclusion into drug formularies. Technology suppliers across all jurisdictions have the option of dissent/appeal and revision of recommendations is taking place either over a standard period of time or when new evidence becomes available.

Our results show that additional value concerns going beyond economic evaluation or clinical benefit assessment are captured to a different extent or included in the evaluation process as criteria that may help to explain some of the heterogeneity observed in coverage recommendations and decision-making.

Overall, all countries assess similar types of evidence; however, the specific endpoints used, their level of provision and requirement, the way they are incorporated (e.g. explicitly vs. implicitly) and their relative importance vary across countries. The same holds for the interpretation of the submitted evidence by HTA agencies [7]. Overall, the main evidence assessed could be divided into six clusters of information: (1) burden of disease, (2) therapeutic and safety impact, (3) innovation level, (4) socioeconomic impact, (5) efficiency considerations, and (6) other sources of evidence and criteria.

### Conceptual and methodological limitations in value assessment

Current value assessment (VA) approaches mainly consider comparative clinical efficacy in combination with clinical cost-effectiveness techniques, while increasingly incorporating real world data after a new drug has entered the market, thus essentially reflecting comparative effectiveness and efficiency. However, there is considerable subjectivity in the criteria selection used to interpret evidence and determine product value, notably which metrics can be used to measure efficacy and effectiveness, what type of costs need to be considered, and, very importantly, how to account for other key dimensions of value.

Most VA approaches examine the efficacy/effectiveness, or cost-effectiveness of new interventions by mostly addressing only a partial dimension of ‘overall value’ in a systematic and explicit manner that relates mainly to ‘scientific value judgments’ (ScVJ) of their therapeutic aspect (e.g. safety, efficacy, effectiveness), possibly in relation to cost. However, as many HTA agencies have recognised (at least indirectly), the value of new medical technologies is multi-dimensional, and not only limited to clinical benefit and cost. In addition to commonly used ScVJ, which are based solely on “scientific” evidence relating to clinical cost-effectiveness and ICERs, other “social” value factors (social value judgements—SoVJ), falling under the information clusters of burden of disease, innovation level and socioeconomic impact, also play a definitive role in the deliberative process and, ultimately, in decision-making; however, there is little, if any, evidence on how SoVJ are captured formally in the appraisal process across settings.

In most settings, the absence of clarity on the use of SoVJ, including their interplay with ScVJ, and their influence on coverage recommendations, remains unknown. SoVJs are usually considered implicitly by HTAs or decision-makers mostly on an ad-hoc basis. In most cases it is not known what their relative importance is, and what trade-offs HTA agencies are willing to make. As a result, the concept of ‘overall value’ remains elusive, given that multiple evaluation criteria apply across different settings, with differential intensity and in a non-systematic manner.

### Policy implications and ways forward

Following the technical review of policy initiatives and opportunities for collaboration and research for access to new medicines in Europe, WHO proposes far more extensive use of HTA in decision-making [91]. However, for this to take place, a more holistic perspective and coordinated action would be needed.

Decision-makers, as well as other stakeholders, need clear, comprehensive and transparent ways of assessing clinical and economic benefit and the impact those new treatments have, from a wider socio-economic perspective, in order to make rational decisions about priority setting. Not having such methods creates a conceptual, methodological and policy gap. Appropriate adaptations of current methodologies, or development of new transparent conceptual frameworks, seem to be needed.

NICE in England is one of the forerunner agencies in acknowledging, formalising and creating a methodological landscape for SoVJ, which include, first, the burden of disease the treatment addresses, hence the clinical and policy importance of the health topic under consideration; second, the cost impact on resources from a societal perspective; third, policy objectives relating to the long-term benefits of innovation [92–94], and, in general, the broader balance between benefits and costs. The existing influence of disease severity could be illustrated in the context of EoL treatments, where QALYs gained for terminal illnesses have a greater weight [95], on the grounds that society places a special value on extending the lives of the terminally ill [96]. Decision makers have been exploring new ways of considering additional value parameters, while highlighting the need for “a broader and more transparent assessment” methodology, suggesting a move towards value-based assessment [97, 98]. A comparable approach highlighting the broader societal implications of introducing a new technology, addressing considerations of need, equity and human dignity, are also present explicitly in the case of the Swedish TLV. Despite the explicit nature of these broader considerations, it is unclear what their influence is in shaping VAs and coverage recommendations.

Aspects of HTA shortcomings have also been reflected by various recent initiatives seeking to establish “value frameworks” aiming to aid pricing and clinical practice decisions by considering a variety of parameters for the assessment of value, possibly in relation to costs. Most of that work has been led by professional associations seeking clarity on the determinants of value and their relative importance to different stakeholders [99–103]. However, attention should be paid to their methodologies, for recommendations to be robust and to avoid misguided decisions [104]. All these initiatives have attempted to adopt multi-criteria evaluation approaches, albeit in a very simplified and relatively abstract manner. Other approaches embedded in decision analysis could address benefit-risk assessment considerations of health care interventions [105, 106]. Considering the limitations highlighted by this systematic review in the context of HTA as it is practised currently, it looks as though multi-criteria decision analysis methods could be explored to capture the value of new medical technologies in a holistic manner and, through this, facilitate HTA decision-making processes in a spirit of transparency, comprehensiveness, and flexibility [107, 108].

The heterogeneity in VA systems across Europe, which also results in significant difference in coverage recommendations across settings based on how HTA agencies perceive or interpret evidence and the associated uncertainties, has recently acquired another important dimension; in September 2016, the European Commission outlined its thoughts to strengthen EU cooperation on HTA [109]. The Commission’s vision includes several options, ranging from voluntary long-term cooperation to cooperation on the production of full joint HTA reports. While it is very premature to speculate what the likely outcome of this initiative is going to be beyond 2020, when the current Joint Action 3 ends, the Commission’s desired course of action seems to be in favour of greater collaboration amongst HTA agencies. Whatever the form of collaboration, member states will undoubtedly contend that the principle of subsidiarity will need to hold. This implies that member states will continue to exercise control on appraisals and coverage recommendations, but assessment could be done through some form of collaborative arrangement (jointly, via mutual recognition, or otherwise). If so, the precise criteria that are acceptable across member states will need to be clarified and explicitly incorporated into the assessment process. The current heterogeneity in coverage recommendations, which results partly from differences in methods applied in the assessment phase, and special considerations/social value judgements applied in the appraisal phase, may need to be addressed by recognising the relative importance of the latter in the assessment phase. This would provide greater steering to member states during the appraisal phase when they seek to make final decisions on coverage. It will also require significant debate in order to come to a joint understanding on the different criteria and their relative importance that can be used in and inform the assessment phase beyond costs and effects.

## Conclusion

The study highlights a number of significant similarities but also considerable differences in the practices, processes and policies of VA for new medicines across eight study countries in Europe. These differences exist because of different national priorities between countries, but also because of different processes and methodological frameworks adopted for the elicitation of decision-makers’ preferences. Overall, there is considerable ambiguity with regards to what additional value criteria to incorporate, how to establish their relative importance, and whose preferences to consider. Currently, all these decisions are subject to decision-makers’ discretion, but are in most cases exemplified in a less than transparent way, potentially resulting in some form of bias.

Procedures characterized by greater transparency or clarity in terms of value criteria used and a higher degree of comprehensiveness and methodological robustness could lead to more rational evidence-based decision making, contributing to more efficient resource allocation and, potentially, higher societal welfare, while also raising public confidence and fairness in terms of homogeneity and consistency of decision outcomes.

The limitations of the current VA methodologies and the identified conceptual and policy gaps suggest that there is a need for methodological approaches that encompass multiple evaluation criteria explicitly, so that value can be an explicit function of a number of dimensions beyond those that are currently explicitly and sytematically captured. This is increasingly becoming imperative in the context of European collaboration, particularly if some form of joint assessment at EU level is likely to emerge beyond 2020. Decision analysis and multi-criteria evaluation approaches could potentially provide the foundation for measuring and eliciting the value of new medicines and technologies as they provide a comprehensive alternative for quantitative modelling.

## Electronic supplementary material

Below is the link to the electronic supplementary material.

Supplementary material 1 (DOCX 110 kb)

## Footnotes

1 Other HTA agencies exist on regional level (e.g. UVEF is responsible for HTAs in the Veneto region).

2 RedETS is the Spanish Network of regional HTA agencies coordinated by Institutde Salud Carlos III (ISCIII) and could be regarded as the National HTA advisory body at federal level. However, at this (federal) level it does not assess pharmaceuticals, but mostly non-drug health technologies, such as screening programmes and medical devices. Although the ICP, led by the Dirección General de Farmacia under the Ministry of Health, is the committee responsible for the assessment of drugs, producing mandatory decisions at federal level regarding the reimbursement and pricing of pharmaceuticals, the vast majority of economic evaluations for drugs are conducted at autonomous community level by regional HTA agencies.
