# Supplementary material for: Using health technology assessment to assess the value of new medicines: results of a systematic review and expert consultation across eight European countries
Source: Eur J Health Econ. 2017 Mar 16;19(1):123–52. doi: 10.1007/s10198-017-0871-0 (PMC5773640; doi:10.1007/s10198-017-0871-0)
Supplement: Supplementary file 1 — Supplementary material 1 (DOCX 110 kb) [file 10198_2017_871_MOESM1_ESM.docx]

Appendix 1

List of articles included in the results identified from the review of the literature (alphabetical order) [1-101]:

References

1. AHTAPol: Guidelines for conducting Health Technology Assessment (HTA). In. Agency for Health Technology Assessment, Warsaw, (2009)

2. AIFA: The Italian Medicines Agency. <http://www.agenziafarmaco.com/en/node/4111>. Accessed June 2016

3. Antoñanzas, F.: Challenges to Achieving Value in Drug Spending in a Decentralized Country: The Spanish Case. Value in Health **6**, S52-S63 (2003). doi:10.1046/j.1524-4733.6.s1.6.x

4. Banta, D., de Wit, A.: Public health services and cost-effectiveness analysis. Annual Review Of Public Health **29**, 383-397 (2008). doi:10.1146/annurev.publhealth.29.020907.090808

5. Banta, D., Oortwijn, W.J.: The Netherlands. International Journal Of Technology Assessment In Health Care **25 Suppl 1**, 143-147 (2009). doi:10.1017/S0266462309090564

6. Bell, K.J.L., Irwig, L., March, L.M., Hayen, A., Macaskill, P., Craig, J.C.: Should response rules be used to decide continued subsidy of very expensive drugs? A checklist for decision makers. Pharmacoepidemiology And Drug Safety **19**(1), 99-105 (2010). doi:10.1002/pds.1868

7. Boersma, C., Broere, A., Postma, M.J.: Quantification of the potential impact of cost-effectiveness thresholds on dutch drug expenditures using retrospective analysis. Value In Health: The Journal Of The International Society For Pharmacoeconomics And Outcomes Research **13**(6), 853-856 (2010). doi:10.1111/j.1524-4733.2010.00736.x

8. Briggs, A.H., Levy, A.R.: Pharmacoeconomics and Pharmacoepidemiology: Curious bedfellows or a match made in heaven? In, vol. 24. pp. 1079-1086. (2006)

9. Bryan, S., Williams, I., McIver, S.: Seeing the NICE side of cost-effectiveness analysis: a qualitative investigation of the use of CEA in NICE technology appraisals. Health Economics **16**(2), 179-193 (2007).

10. Buxton, M.: Implications of the appraisal function of the National Institute for Clinical Excellence (NICE). Value In Health: The Journal Of The International Society For Pharmacoeconomics And Outcomes Research **4**(3), 212-216 (2001).

11. Calltorp, J.: Priority setting in health policy in Sweden and a comparison with Norway. Health Policy (Amsterdam, Netherlands) **50**(1-2), 1-22 (1999).

12. Capri, S., Ceci, A., Terranova, L., Merlo, F., Mantovani, L.: Guidelines for economic evaluations in Italy: Recommendations from the Italian Group of Pharmacoeconomic Studies. Drug Information Journal **35**, 189-201 (2001).

13. Catalá-López, F., Alonso-Arroyo, A., Aleixandre-Benavent, R., Ridao, M., Bolaños, M., García-Altés, A., Sanfélix-Gimeno, G., Peiró, S.: Coauthorship and institutional collaborations on cost-effectiveness analyses: a systematic network analysis. Plos One **7**(5), e38012-e38012 (2012). doi:10.1371/journal.pone.0038012

14. Chalkidou, K.: Evidence and values: paying for end-of-life drugs in the British NHS. Health Economics, Policy, And Law **7**(4), 393-409 (2012). doi:10.1017/S1744133112000205

15. Cleemput, I., Franken, M., Koopmanschap, M., le Polain, M.: European drug reimbursement systems' legitimacy: five-country comparison and policy tool. International Journal Of Technology Assessment In Health Care **28**(4), 358-366 (2012). doi:10.1017/S0266462312000529

16. Clement, F.M., Harris, A., Li, J.J., Yong, K., Lee, K.M., Manns, B.J.: Using effectiveness and cost-effectiveness to make drug coverage decisions: a comparison of Britain, Australia, and Canada. JAMA **302**(13), 1437-1443 (2009). doi:10.1001/jama.2009.1409

17. Cookson, R., McDaid, D., Maynard, A.: Wrong SIGN, NICE mess: is national guidance distorting allocation of resources? BMJ (Clinical Research Ed.) **323**(7315), 743-745 (2001).

18. Corbacho, B., Pinto-Prades, J.L.: Health economic decision-making: a comparison between UK and Spain. British Medical Bulletin **103**(1), 5-20 (2012). doi:10.1093/bmb/lds017

19. CVZ: Guidelines for pharmacoeconomic research, updated version. In. College voor zorgverzekeringen, (2006)

20. Dakin, H.A., Devlin, N.J., Odeyemi, I.A.O.: “Yes”, “No” or “Yes, but”? Multinomial modelling of NICE decision-making. Health Policy **77**(3), 352-367 (2006). doi:10.1016/j.healthpol.2005.08.008

21. Devlin, N., Parkin, D.: Does NICE have a cost-effectiveness threshold and what other factors influence its decisions? A binary choice analysis. Health Economics **13**(5), 437-452 (2004).

22. DH: Impact Assessment of Proposal for a Cancer Drug Fund. In: Health, D.o. (ed.). (2010)

23. Dirksen, C.D., Utens, C.M., Joore, M.A., van Barneveld, T.A., Boer, B., Dreesens, D.H., van Laarhoven, H., Smit, C., Stiggelbout, A.M., van der Weijden, T.: Integrating evidence on patient preferences in healthcare policy decisions: protocol of the patient-VIP study. Implementation Science: IS **8**, 64-64 (2013). doi:10.1186/1748-5908-8-64

24. Drummond, M., Jönsson, B., Rutten, F., Stargardt, T.: Reimbursement of pharmaceuticals: reference pricing versus health technology assessment. The European Journal Of Health Economics: HEPAC: Health Economics In Prevention And Care **12**(3), 263-271 (2011). doi:10.1007/s10198-010-0274-y

25. Falissard, B., Izard, V., Xerri, B., Bouvenot, G., Meyer, F., Degos, L.: Relative effectiveness assessment of listed drugs (REAL): a new method for an early comparison of the effectiveness of approved health technologies. International Journal Of Technology Assessment In Health Care **26**(1), 124-130 (2010). doi:10.1017/S0266462309990821

26. Favaretti, C., Cicchetti, A., Guarrera, G., Marchetti, M., Ricciardi, W.: Health technology assessment in Italy. International Journal Of Technology Assessment In Health Care **25 Suppl 1**, 127-133 (2009). doi:10.1017/S0266462309090539

27. Folino-Gallo, P., Montilla, S., Bruzzone, M., Martini, N.: Pricing and reimbursement of pharmaceuticals in Italy. The European Journal Of Health Economics: HEPAC: Health Economics In Prevention And Care **9**(3), 305-310 (2008). doi:10.1007/s10198-008-0114-5

28. Franken, M., le Polain, M., Cleemput, I., Koopmanschap, M.: Similarities and differences between five European drug reimbursement systems. International Journal Of Technology Assessment In Health Care **28**(4), 349-357 (2012). doi:10.1017/S0266462312000530

29. Franken, M., Nilsson, F., Sandmann, F., Boer, A., Koopmanschap, M.: Unravelling Drug Reimbursement Outcomes: A Comparative Study of the Role of Pharmacoeconomic Evidence in Dutch and Swedish Reimbursement Decision Making. PharmacoEconomics **31**(9), 781-797 (2013). doi:10.1007/s40273-013-0074-1

30. Freemantle, N.: Biostatistical aspects for the use of evidence based medicine in health technology assessment. The European Journal Of Health Economics: HEPAC: Health Economics In Prevention And Care **9 Suppl 1**, 31-43 (2008). doi:10.1007/s10198-008-0123-4

31. Gafni, A., Birch, S.: NICE Methodological Guidelines and Decision Making in the National Health Service in England and Wales. PharmacoEconomics **21**(3), 149-157 (2003).

32. Golan, O., Hansen, P., Kaplan, G., Tal, O.: Health technology prioritization: Which criteria for prioritizing new technologies and what are their relative weights? Health policy **102**(2), 126-135 (2011). doi:10.1016/j.healthpol.2010.10.012

33. Gridchyna, I., Aulois-Griot, M., Maurain, C., Bégaud, B.: How innovative are pharmaceutical innovations?: the case of medicines financed through add-on payments outside of the French DRG-based hospital payment system. Health Policy (Amsterdam, Netherlands) **104**(1), 69-75 (2012). doi:10.1016/j.healthpol.2011.11.007

34. HAS: General method for assessing health technologies. In. Haute Autorité de Santé, (2007)

35. HAS: Choices in Methods for Economic Evaluation. In. Haute Autorité de santé, (2012)

36. HAS: La Commission évaluation économique et de santé publique (CEESP). In. Haute Autorité de Santé, (2012)

37. HAS: Évaluation médico-économique des produits de santé. <http://www.has-sante.fr/portail/jcms/r_1502595/fr/evaluation-medico-economique-des-produits-de-sante> (2013). Accessed June 2016

38. Hjalte, K., Hjelmgren, J., Johansson, F., Persson, U.: Betalningsviljan for ett kvalitetsjusterat levnadsar - en pilotstudie. In. IHE, (2005)

39. Hoyle, M.: Historical lifetimes of drugs in England: application to value of information and cost-effectiveness analyses. Value In Health: The Journal Of The International Society For Pharmacoeconomics And Outcomes Research **13**(8), 885-892 (2010). doi:10.1111/j.1524-4733.2010.00778.x

40. Hughes-Wilson, W., Palma, A., Schuurman, A., Simoens, S.: Paying for the Orphan Drug System: break or bend? Is it time for a new evaluation system for payers in Europe to take account of new rare disease treatments? Orphanet Journal Of Rare Diseases **7**, 74-74 (2012). doi:10.1186/1750-1172-7-74

41. IQWiG: General Methods for the Assessment of the Relation of Benefits to Costs. In. Institute for Quality and Efficiency in Health Care, Cologne, (2009)

42. IQWiG: General Methods. In. Institute for Quality and Efficiency in Health Care, Cologne, (2011)

43. ISPOR: ISPOR Global Health Care Systems Road Map - Spain. <http://www.ispor.org/htaroadmaps/spain.asp> (2009). Accessed June 2016

44. ISPOR: Pharmacoeconomic Guidelines Around the World. <http://www.ispor.org/peguidelines/index.asp> (2014). Accessed June 2016

45. Jena, A.B., Philipson, T.J.: Endogenous cost-effectiveness analysis and health care technology adoption. Journal Of Health Economics **32**(1), 172-180 (2013). doi:10.1016/j.jhealeco.2012.10.002

46. Jönsson, B.: Economic evaluation for pharmaceuticals in Germany. The European Journal Of Health Economics: HEPAC: Health Economics In Prevention And Care **8 Suppl 1**, S1-S2 (2007).

47. Kaltenthaler, E., Boland, A., Carroll, C., Dickson, R., Fitzgerald, P., Papaioannou, D.: Evidence Review Group approaches to the critical appraisal of manufacturer submissions for the NICE STA process: a mapping study and thematic analysis. Health Technology Assessment (Winchester, England) **15**(22), 1 (2011). doi:10.3310/hta15220

48. Kenny, N., Joffres, C.: An ethical analysis of international health priority-setting. Health Care Analysis: HCA: Journal Of Health Philosophy And Policy **16**(2), 145-160 (2008). doi:10.1007/s10728-007-0065-5

49. Kirkdale, R., Krell, J., Hanlon Brown, C., Tuthill, M., Waxman, J.: The cost of a QALY. QJM: An International Journal of Medicine **103**(9), 715-720 (2010). doi:10.1093/qjmed/hcq081

50. Kleijnen, S., George, E., Goulden, S., d'Andon, A., Vitré, P., Osińska, B., Rdzany, R., Thirstrup, S., Corbacho, B., Nagy, B.Z., Leufkens, H.G., de Boer, A., Goettsch, W.G.: Relative effectiveness assessment of pharmaceuticals: similarities and differences in 29 jurisdictions. Value In Health: The Journal Of The International Society For Pharmacoeconomics And Outcomes Research **15**(6), 954-960 (2012). doi:10.1016/j.jval.2012.04.010

51. Kolasa, K., Schubert, S., Manca, A., Hermanowski, T.: A review of Health Technology Assessment (HTA) recommendations for drug therapies issued between 2007 and 2009 and their impact on policymaking processes in Poland. Health Policy **102**(2/3), 145-151 (2011). doi:10.1016/j.healthpol.2011.05.001

52. le Pen, C.: Pharmaceutical economy and the economic assessment of drugs in France. Social Science & Medicine (1982) **45**(4), 635-643 (1997).

53. Le Pen, C., Priol, G., Lilliu, H.: What criteria for pharmaceuticals reimbursement? An empirical analysis of the evaluation of "medical service rendered" by reimbursable drugs in France. The European Journal Of Health Economics: HEPAC: Health Economics In Prevention And Care **4**(1), 30-36 (2003).

54. López-Bastida, J., Oliva, J., Antoñanzas, F., García-Altés, A., Gisbert, R., Mar, J., Puig-Junoy, J.: Spanish recommendations on economic evaluation of health technologies. The European Journal Of Health Economics: HEPAC: Health Economics In Prevention And Care **11**(5), 513-520 (2010). doi:10.1007/s10198-010-0244-4

55. Manchikanti, L., Falco, F.J.E., Boswell, M.V., Hirsch, J.A.: Facts, fallacies, and politics of comparative effectiveness research: Part 2 - implications for interventional pain management. Pain Physician **13**(1), E55-E79 (2010).

56. Mangiapane, S., Velasco Garrido, M.: Use of Surrogate end points in HTA. GMS Health Technology Assessment **5**, Doc12-Doc12 (2009). doi:10.3205/hta000074

57. Mapelli, V., Lucioni, C.: Spending on pharmaceuticals in Italy: macro constraints with local autonomy. Value In Health: The Journal Of The International Society For Pharmacoeconomics And Outcomes Research **6 Suppl 1**, S31-S45 (2003).

58. Mason, A.R., Drummond, M.F.: Public funding of new cancer drugs: Is NICE getting nastier? European Journal Of Cancer (Oxford, England: 1990) **45**(7), 1188-1192 (2009). doi:10.1016/j.ejca.2008.11.040

59. Mauskopf, J.A., Sullivan, S.D., Annemans, L., Caro, J., Mullins, C.D., Nuijten, M., Orlewska, E., Watkins, J., Trueman, P.: Principles of good practice for budget impact analysis: report of the ISPOR Task Force on good research practices--budget impact analysis. Value In Health: The Journal Of The International Society For Pharmacoeconomics And Outcomes Research **10**(5), 336-347 (2007).

60. Mauskopf, J., Walter, J., Birt, J., Bowman, L., Copley-Merriman, C., Drummond, M.: Differences among formulary submission guidelines: implications for health technology assessment. International Journal Of Technology Assessment In Health Care **27**(3), 261-270 (2011). doi:0.1017/S0266462311000274

61. McGhan, W.F., Al, M., Doshi, J.A., Kamae, I., Marx, S.E., Rindress, D.: The ISPOR Good Practices for Quality Improvement of Cost-Effectiveness Research Task Force Report. Value In Health: The Journal Of The International Society For Pharmacoeconomics And Outcomes Research **12**(8), 1086-1099 (2009). doi:10.1111/j.1524-4733.2009.00605.x

62. McMahon, M., Morgan, S., Mitton, C.: The Common Drug Review: A NICE start for Canada? Health Policy **77**(3), 339-351 (2006). doi:10.1016/j.healthpol.2005.08.006

63. Milne, R., Clegg, A., Stevens, A.: HTA responses and the classic HTA report. Journal Of Public Health Medicine **25**(2), 102-106 (2003).

64. Morgan, S.G., McMahon, M., Mitton, C., Roughead, E., Kirk, R., Kanavos, P., Menon, D.: Centralized drug review processes in Australia, Canada, New Zealand, and the United kingdom. Health Affairs (Project Hope) **25**(2), 337-347 (2006).

65. NICE: SOCIAL VALUE JUDGEMENTS: Principles for the development of NICE guidance. In. National Institute for Health and Care Excellence, London, (2008)

66. NICE: The guidelines manual. In. National Institute for Health and Clinical Excellence, (2009)

67. NICE: Guide to the methods of technology appraisal 2013. In. National Institute for Health and Care Excellence, (2013)

68. NICE: Interim Process and Methods of the Highly Specialised Technologies Programme. In. National Institute for Health and Care Excellence, (2013)

69. Niżankowski, R., Wilk, N.: From idealistic rookies to a regional leader: The history of health technology assessment in Poland. International Journal of Technology Assessment in Health Care **25**(S1), 156-162 (2009). doi:10.1017/S0266462309090588

70. Oostenbrink, J.B., Koopmanschap, M.A., Rutten, F.F.H.: Standardisation of costs: The Dutch Manual for Costing in Economic Evaluations. PharmacoEconomics **20**(7), 443-454 (2002).

71. Ozieranski, P., McKee, M., King, L.: The politics of health technology assessment in Poland. Health policy **108**(2-3), 178-193 (2012). doi:10.1016/j.healthpol.2012.10.001

72. Persson, U., Hjelmgren, J.: Healso-och sjukvarden behover kunskap om hur befolkningen varderar halsan. Lakartidningen **100**(43), 3436–3437 (2003).

73. Persson, U., Willis, M., Ödegaard, K.: A case study of ex ante , value- based price and reimbursement decision- making: TLV and rimonabant in Sweden. Eur J Health Econ **11**(2), 195-203 (2010). doi:10.1007/s10198-009-0166-1

74. Raftery, J.: Review of NICE'S recommendations, 1999- 2005. British Medical Journal **332**(7552), 1266-1268 (2006).

75. Rawlins, M., Culyer, A.: National institute for clinical excellence and its value judgments. British Medical Journal **329**(7459), 224-227 (2004).

76. Riedel, R., Repschläger, U., Griebenow, R., Breitkopf, S., Schmidt, S., Guhl, A.: International standards for health economic evaluation with a focus on the German approach. Journal Of Clinical Pharmacy And Therapeutics **38**(4), 277-285 (2013). doi:10.1111/jcpt.12043

77. Rodríguez-Monguío, R., Villar, F.A.: Healthcare Rationing in Spain: Framework, Descriptive Analysis and Consequences. PharmacoEconomics **24**(6), 537-548 (2006).

78. Rodriguez, J.M., Paz, S., Lizan, L., Gonzalez, P.: The use of quality-adjusted life-years in the economic evaluation of health technologies in Spain: a review of the 1990-2009 literature. Value In Health: The Journal Of The International Society For Pharmacoeconomics And Outcomes Research **14**(4), 458-464 (2011). doi:10.1016/j.jval.2010.10.039

79. Rogowski, W.H., Hartz, S.C., John, J.H.: Clearing up the hazy road from bench to bedside: a framework for integrating the fourth hurdle into translational medicine. BMC Health Services Research **8**, 194-194 (2008). doi:10.1186/1472-6963-8-194

80. Sabik, L.M., Lie, R.K.: Priority setting in health care: Lessons from the experiences of eight countries. International Journal For Equity In Health **7**, 4-4 (2008). doi:10.1186/1475-9276-7-4

81. Schlander, M.: Is NICE infallible? A qualitative study of its assessment of treatments for attention-deficit/hyperactivity disorder (ADHD). Current Medical Research And Opinion **24**(2), 515-535 (2008). doi:10.1185/030079908X260808

82. Schubert, F.: Health technology assessment. The pharmaceutical industry perspective. International Journal Of Technology Assessment In Health Care **18**(2), 184-191 (2002).

83. Sheldon, T.A., Cullum, N., Dawson, D., Lankshear, A., Lowson, K., Watt, I., West, P., Wright, D., Wright, J.: What's the evidence that NICE guidance has been implemented? Results from a national evaluation using time series analysis, audit of patients' notes, and interviews. BMJ (Clinical Research Ed.) **329**(7473), 999-999 (2004).

84. Simoens, S.: Use of economic evaluation in decision making: evidence and recommendations for improvement. Drugs **70**(15), 1917-1926 (2010). doi:10.2165/11538120-000000000-00000

85. Sjögren, E.: Deciding subsidy for pharmaceuticals based on ambiguous evidence. Journal Of Health Organization And Management **22**(4), 368-383 (2008).

86. Stewart, A., Aubrey, P., Belsey, J.: Addressing the health technology assessment of biosimilar pharmaceuticals. Current Medical Research And Opinion **26**(9), 2119-2126 (2010). doi:10.1185/03007995.2010.505137

87. Stolk, E.A., de Bont, A., van Halteren, A.R., Bijlmer, R.J., Poley, M.J.: Role of health technology assessment in shaping the benefits package in The Netherlands. Expert Review Of Pharmacoeconomics & Outcomes Research **9**(1), 85-94 (2009). doi:10.1586/14737167.9.1.85

88. Stoykova, B., Drummond, M., Barbieri, M., Kleijnen, J.: The lag between effectiveness and cost-effectiveness evidence of new drugs. Implications for decision-making in health care. The European Journal Of Health Economics: HEPAC: Health Economics In Prevention And Care **4**(4), 313-318 (2003).

89. Sun, F., Schoelles, K.: A systematic review of methods for health care technology horizon scanning. In: AHRQ Publication No. 13-EHC104-EF. Agency for Healthcare Research and Quality, Rockville, MD, (2013)

90. Committee, T.U.K.H.o.C.H.: National Institute for Clinical Excellence: Second Report of Session 2001–2002. In., vol. HC 515. (2002)

91. TLV: Act (2002:160) on Pharmaceutical Benefits, etc. In. The Dental and Pharmaceutical Benefits Agency, (2002)

92. TLV: Guide for companies when applying for subsidies and pricing for pharmaceutical products. In. The Dental and Pharmaceutical Benefits Agency, (2012)

93. TLV: Handbook – reviewing the reimbursement status of pharmaceuticals. In. The Dental and Pharmaceutical Benefits Agency, (2012)

94. Towse, A., Pritchard, C.: National Institute for Clinical Excellence (NICE): Is Economic Appraisal Working? PharmacoEconomics **20**(15), 95-105 (2002).

95. Trowman, R., Chung, H., Longson, C., Littlejohns, P., Clark, P.: The National Institute for Health and Clinical Excellence and its role in assessing the value of new cancer treatments in England and Wales. Clinical Cancer Research: An Official Journal Of The American Association For Cancer Research **17**(15), 4930-4935 (2011). doi:10.1158/1078-0432.CCR-10-2510

96. Van Oostenbruggen, M.F., Jansen, R.B., Mur, K., Kooijman, H.: Penny and pound wise: Pharmacoeconomics from a governmental perspective. PharmacoEconomics **23**(3), 219-226 (2005). doi:10.2165/00019053-200523030-00003

97. Van Wilder, P.B., Bormans, V.V., Dupont, A.G.: Relative efficacy and effectiveness assessment of new pharmaceuticals in three EU member states: current practices and outcome agreement between Belgium, the Netherlands and France. European Journal Of Clinical Pharmacology **69**(12), 2037-2043 (2013). doi:10.1007/s00228-013-1577-6

98. von der Schulenburg, J.M.G., Vauth, C., Mittendorf, T., Greiner, W.: Methods for determining cost-benefit ratios for pharmaceuticals in Germany. The European Journal Of Health Economics: HEPAC: Health Economics In Prevention And Care **8 Suppl 1**, S5-S31 (2007).

99. Weill, C., Banta, D.: Development of health technology assessment in France. International Journal Of Technology Assessment In Health Care **25 Suppl 1**, 108-111 (2009). doi:10.1017/S0266462309090503

100. Zentner, A., Velasco-Garrido, M., Busse, R.: Methods for the comparative evaluation of pharmaceuticals. GMS Health Technology Assessment **1**, Doc09-Doc09 (2005).

101. ZIN: ZIN homepage. <http://www.zorginstituutnederland.nl/>. Accessed June 2016

Appendix 2

List of articles included in the results identified during the expert consultation process (alphabetical order) [1-5]

References

1. Claxton, K., Martin, S., Soares, M., Rice, N., Spackman, E., Hinde, S., Devlin, N., Smith, P.C., Sculpher, M.: Methods for the estimation of the National Institute for Health and care excellence cost- effectiveness threshold. Health Technology Assessment **19**(14), 1-503 (2015). doi:10.3310/hta19140

2. G-BA: Chapter 5: Benefit Assessment of pharmaceuticals according to s. 35a SGB V. In. Gemeinsame Bundesausschuss, (2015)

3. IQWiG: General Methods. In. Institut für Qualität und Wirtschaftlichkeit im Gesundheitswesen, (2015)

4. Svensson, M., Nilsson, F., Arnberg, K.: Reimbursement Decisions for Pharmaceuticals in Sweden: The Impact of Disease Severity and Cost Effectiveness. PharmacoEconomics **33**(11), 1229-1236 (2015). doi:10.1007/s40273-015-0307-6

5. Toumi, M., Rémuzat, C., El Hammi, E., Millier, A., Aballéa, S., Chouaid, C., Falissard, B.: Current process and future path for health economic assessment of pharmaceuticals in France. Journal Of Market Access & Health Policy **3** (2015). doi:10.3402/jmahp.v3.27902
